# The course of mechanical allodynia differs between forelimb innervation territories following median nerve injury in the rat

**DOI:** 10.3389/fnins.2025.1602524

**Published:** 2025-09-19

**Authors:** Jana Ritter, Cosima Prahm, Manuela Büttcher, Thomas V. Wuttke, Adrien Daigeler, Henrik Lauer, Johannes C. Heinzel, Jonas Kolbenschlag

**Affiliations:** ^1^Department of Hand, Plastic, Reconstructive and Burn Surgery, BG Klinik Tübingen, University of Tübingen, Tübingen, Germany; ^2^Veterinary Service and Laboratory Animal Science, Facility for Animal Welfare, University of Tübingen, Tübingen, Germany; ^3^Department of Neurology and Epileptology, Hertie Institute for Clinical Brain Research, University of Tübingen, Tübingen, Germany; ^4^Department of Neurosurgery, University of Tübingen, Tübingen, Germany; ^5^Ludwig Boltzmann Institute for Traumatology - The Research Center in Cooperation with AUVA, Vienna, Austria; ^6^Austrian Cluster for Tissue Regeneration, Vienna, Austria

**Keywords:** median nerve injury, nerve regeneration, epineurial repair, gait behavior, grasping strength, mechanical allodynia

## Abstract

Functional deficits and chronic neuropathic pain after peripheral nerve injuries pose major clinical challenges. For preclinical evaluation of novel treatments, reliable methods for assessing functional recovery and robust animal models with high translational potential are crucial. Following peripheral nerve injury reinnervation of denervated target organs is not only achieved by regeneration of the original nerve, but also by collateral sprouting of adjacent intact nerves. In this study motor and sensory recovery was analyzed in a rat model of median nerve injury and repair using distinct functional test. Ten male Wistar rats underwent bilateral median nerve transection. In one forelimb the nerve was repaired using epineurial sutures, while in the contralateral forelimb the nerve remained unrepaired and served as internal control. For 12 weeks post-surgery, the Von Frey Test, the Grasping Test and CatWalk gait analysis were performed weekly. Sensory reinnervation and mechanical allodynia were evaluated with the Von Frey Test in distinct predefined test areas considering the innervation of the forepaws, allowing for indirect assessment of collateral nerve sprouting. Early mechanical allodynia developed within two weeks after median nerve injury in all innervation areas of the forepaw. This was associated with significant alterations of Print Width, Print Area, Duty Cycle, Swing Speed, Swing Time and Stand Index. From week 6 onward late mechanical allodynia paralleled with gait improvements and return of measurable grasping strength on the reconstructed side. The methodology utilized in our study, is feasible to comprehensively assess motor and sensory nerve regeneration paving the way for valid evaluation of future therapeutic strategies in a rat median nerve injury model.

## 1 Introduction

Peripheral nerve injuries predominantly affect the upper extremity with the median nerve being the most frequently injured major nerve of the upper extremity (Aman et al., 2022; Karsy et al., 2019). Gold standard treatment for nerve transections is the tension-free direct epineurial repair (Dahlin and Wiberg, 2017; Bellaire et al., 2021; Kubiak et al., 2018). However, only 52% of patients achieve satisfactory motor and 43% regain satisfactory sensory function after median or ulnar nerve injury and repair (Ruijs et al., 2005). Besides, persistent functional deficits, patients are prone to develop chronic neuropathic pain associated with reduced quality of life, sleep disturbances and depression (Colloca et al., 2017; Attal et al., 2011; Finnerup et al., 2016; Dahlin and Wiberg, 2017; Miclescu et al., 2019; Heinzel et al., 2021). However, current pharmacotherapies have limited success rates and are associated with numerous adverse, dose limiting effects (Butler et al., 2019). This highlights the necessity for preclinical research to develop novel therapeutic strategies to enhance functional nerve regeneration and prevent or treat neuropathic pain. Nevertheless, for the evaluation of emerging therapies, robust animal experimental models and reliable assessment methods are essential (Ronchi et al., 2019). Over the past decades, the rat median nerve injury model has gained increasing interest in the scientific community. In this model, limb function is only partially impaired as the intact ulnar and radial nerves continue to facilitate motor and sensory functions within the forelimb (Ronchi et al., 2019). Moreover, automutulatory behavior and joint contractures are less frequent compared to the sciatic nerve model, thus alleviating the evaluation of functional recovery (Haselbach et al., 2016; Weber et al., 1993; Bertelli and Mira, 1995; Ronchi et al., 2019; Papalia et al., 2003; Bontioti et al., 2003). It is further characterized by the availability of functional test such as the Grasping Test (Bertelli and Mira, 1995; Papalia et al., 2003) or the Staircase Test (Montoya et al., 1991; Pagnussat Ade et al., 2009) enabling precise assessment of median nerve function. Several studies further demonstrated the feasibility of CatWalk gait analysis to assess motor and sensory recovery as well as non-evoked pain behaviors and the Von Frey Test to examine mechanical allodynia (Galtrey and Fawcett, 2007; Casal et al., 2020; Heinzel et al., 2022; Heinzel et al., 2020c; Heinzel et al., 2020a; Huerta et al., 2024; Vieira et al., 2020; De La Puente et al., 2022). Nevertheless, while significant correlations between the CatWalk gait analysis and the Grasping Test were identified in the median nerve model (Heinzel et al., 2020c), studies correlating sensory and motor functions in this upper extremity model are still lacking. It should be noted that after nerve injury, reinnervation of target organs can be achieved by regeneration of the original nerve and collateral sprouting from nearby undamaged nerves (Navarro et al., 2007; Jeon et al., 2024; Lemaitre and Court, 2021; Freeman et al., 2020). Although collateral sprouting has long been recognized in humans and animals (Ahčan et al., 1998; Bajrović and Sketelj, 1996; Murray and Thompson, 1957; Nageotte, 1906; Pollock, 1919; Weddell et al., 1941), it is often neglected when evaluating sensory recovery. This is particularly relevant in studies utilizing the Von Frey Test, where first responses to mechanical stimuli after nerve injury are typically attributed to axonal regeneration. The influence of collateral sprouting on sensory reinnervation as assessed with the von Frey Test as well as its significant contribution to the development and maintenance of neuropathic pain has been demonstrated in the sciatic nerve injury model (Cobianchi et al., 2014). To date, the Von Frey Test has not been used to assess collateral sprouting in an upper extremity nerve injury model.

Accordingly, we conducted a pilot study aiming to assess the course of motor and sensory recovery in a median nerve injury and repair model for the first time combining the Von Frey Test, CatWalk (CW) gait analysis and The Grasping Test. The secondary objective was to elucidate correlations between these behavioral tests. Additionally, we aimed to investigate sensory reinnervation across the distinct intervention territories of the forepaws using the Von Frey Test, allowing for functional evaluation of the contribution of collateral sprouting of the ulnar nerve following median nerve injury.

## 2 Materials and methods

### 2.1 Animals and housing

All experimental procedures were approved beforehand by the regional authorities (Regierungspräsidium Tübingen) and followed the Helsinki Declaration on Animal Rights and the Guide for the Care and Use of laboratory animals of the National Institutes of Health. Ten 7–9 weeks old male Wistar rats (Charles River Laboratory, Sulzfeld), weighing approximately 300 g, were used. Prior to any experimental handling, the rats were acclimatized to the new surrounding for 2 week.

### 2.2 Surgical procedure

**FIGURE 1:**
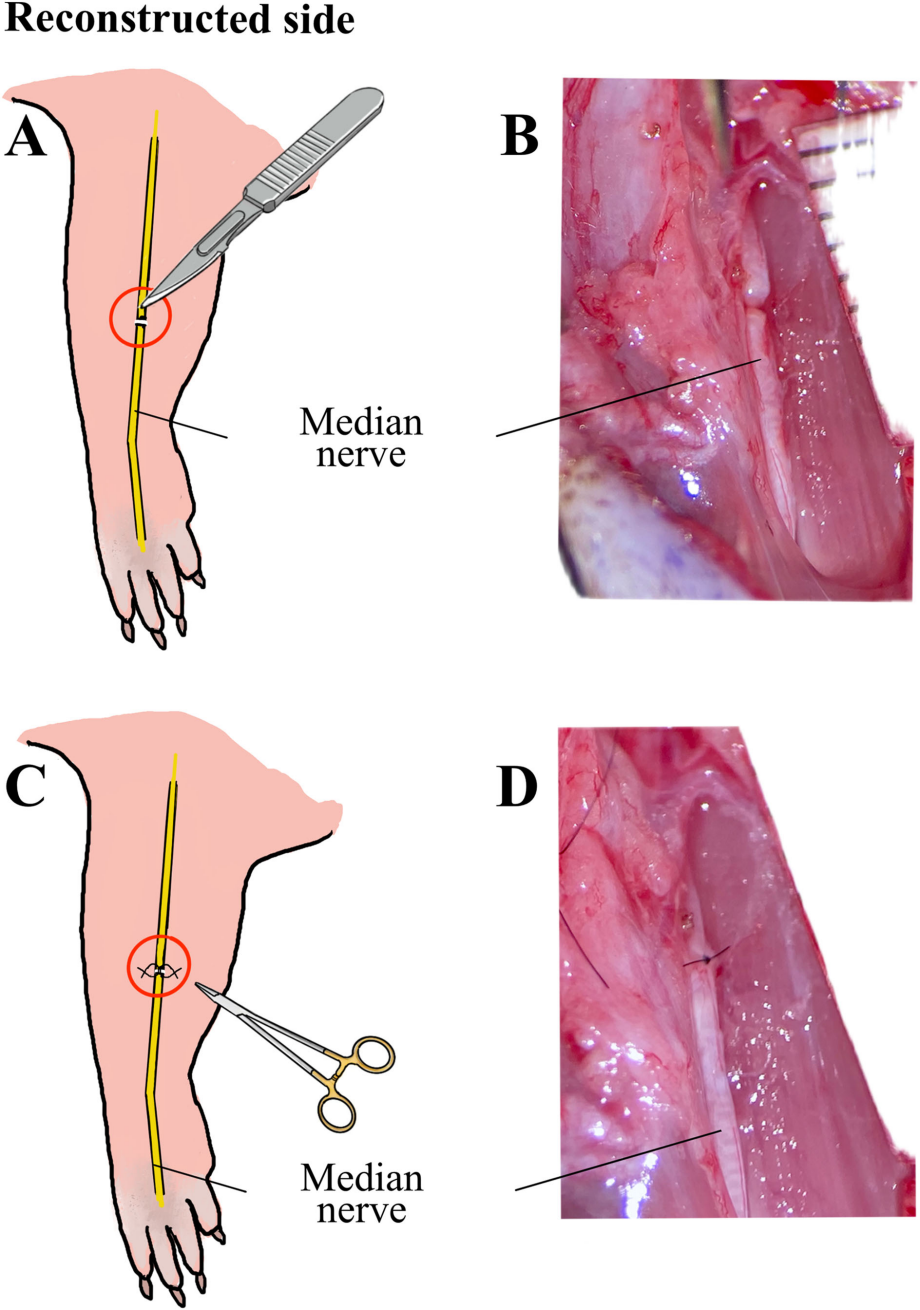
Schematic illustration and intraoperative images of the median nerve injury and repair on the reconstructed side. Schematic illustration **(A)** and intraoperative microscopic view **(B)** of the transection injury of the median nerve on the reconstructed side, as well as schematic illustration **(C)** and microscopic appearance **(D)** of the direct epineurial repair of the median nerve using 10-0 sutures.

**FIGURE 2:**
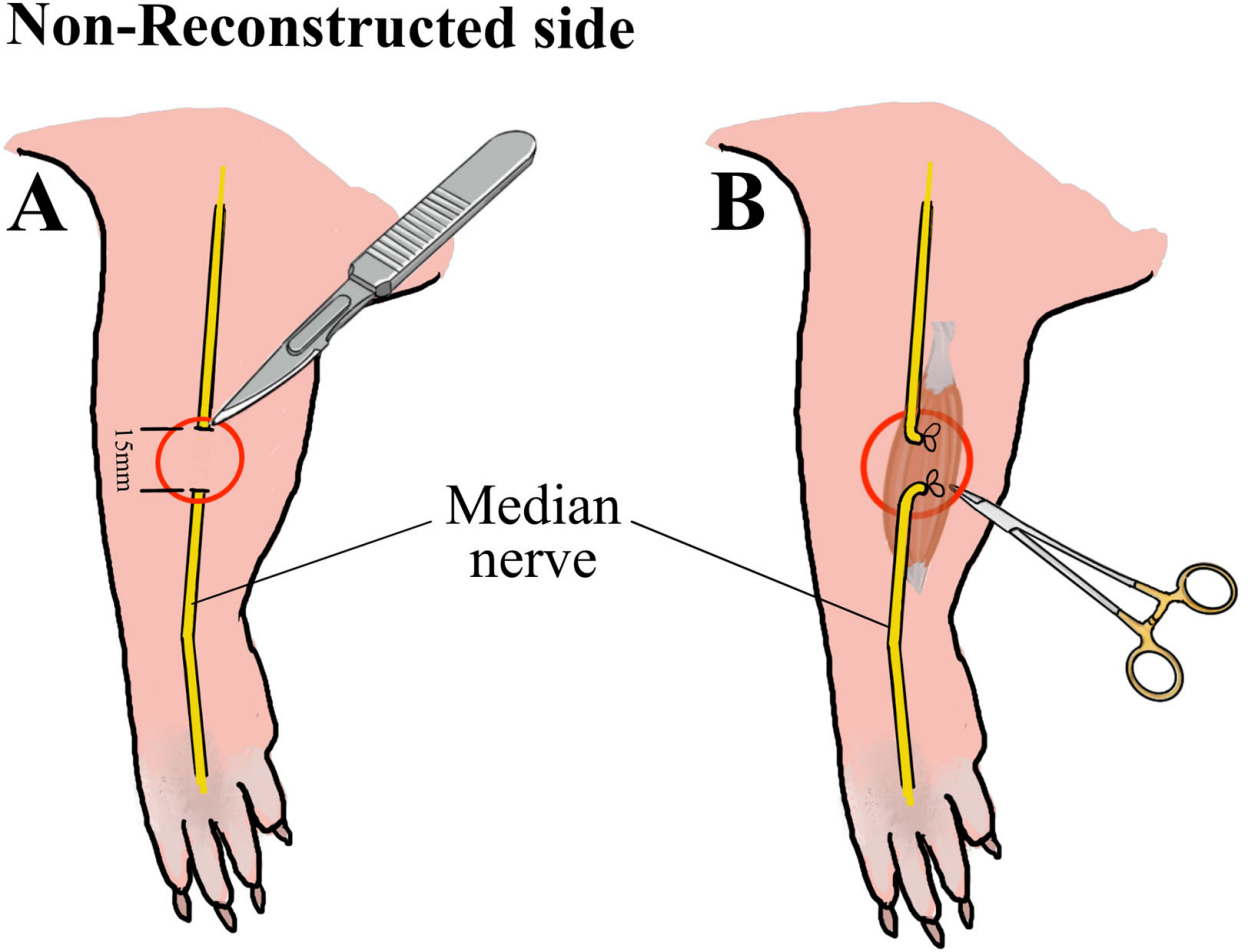
Schematic illustration of the median nerve injury on the non-reconstructed side. Schematic illustration of the resection of 15 mm of the median nerve **(A)** on the non-reconstructed side as well as of the suturing of the proximal and distal nerve stumps into the muscle **(B)** to prevent nerve regeneration.

Approximately 60 min before surgery animals received a subcutaneous injection of Buprenorphine (0.05 mg/kg body weight diluted in 200 μl isotonic Natriumchlorid solution). Anesthesia was induced with 5% Isofluran-oxygen mixture and maintained with 2–3% Isofluran via an open mask system. An ophthalmic ointment was applied to the eyes to prevent drying. Rats were placed on a heated plate to maintain adequate body temperature and breathing, heart rate, temperature and oxygen saturation were continuously monitored during surgical procedures. Ten male Wistar rats underwent bilateral surgery of the median nerve using an operation microscope (Wild M65, Wild Heerbrugg, Switzerland). Reconstruction and control side were randomly assigned for each animal. In one forelimb (reconstructed side), the median nerve was transected (Figure 1A, B) approximately 7 mm proximal to the crossing of the brachial artery and vein and immediately reconstructed using 10-0 epineurial sutures (Ethilon^®^ 10-0, Ethicon-Johnson & Johnson, Brussels, Belgium) (Figure 1C, D). In the other forelimb (non-reconstructed side) a 15-mm segment of the median nerve was resected (Figure 2A) and the nerve stumps were sutured (Ethilon^®^ 10-0, Ethicon-Johnson & Johnson, Brussels, Belgium) into the biceps muscle to prevent spontaneous regeneration (Figure 2B). To provide sufficient analgesia postoperatively, buprenorphine administration (0.05 mg/kg body weight diluted in 200 μl isotonic Natriumchlorid solution) was continued for at least 48 h after surgery in 8 h intervals. Postoperatively, functional nerve regeneration was assessed for 12 weeks. After 12 weeks, rats were euthanized under deep isoflurane anesthesia by intracardial application of an overdose of Thiopental sodium (Thiopental Inresa 0.5 g Inresa Arzneimittel GmbH). Following euthanasia, the flexor digitorum superficialis muscles (FDS) of both sides were harvested and weighed.

### 2.3 Functional testing

Mechanical allodynia, grasping strength and gait behavior were assessed in all rats (*n* = 10) preoperatively and weekly for 12 weeks after surgery by means of the Von Frey Test, the Grasping Test and CatWalk gait analysis (CW). Functional tests were carried out by the same experimenter. With the exception of the Grasping Test, where only the reconstructed side was examined, the experimenter was blind regarding the assignment of the reconstructed and the non-reconstructed side. One week prior to surgeries rats were habituated to the distinct functional test procedures.

#### 2.3.1 Von Frey test

**FIGURE 3:**
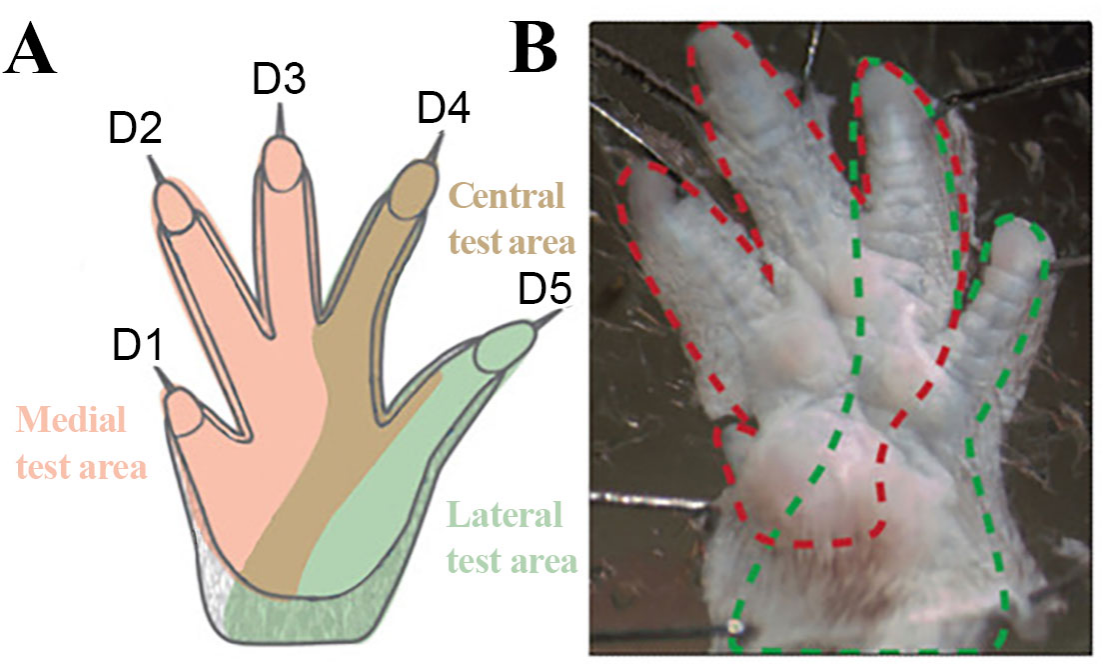
Innervation of the forepaws of the rat. Schematic Illustration **(A)** of the specific predefined test areas considering the sensory innervation of the forepaw and illustration **(B)** of the innervation territories on the glabrous skin of the rat. Rose: Medial test area (innervated by the median nerve); Brown: Central test area (non-selectively innervated by the median and ulnar nerve); Green: Lateral test area (innervated by ulnar nerve). Red dotted line: median nerve innervated areas; Green dotted lines: Ulnar nerve innervated territories. Modified after Walcher et al. (2018).

Sensory reinnervation and mechanical allodynia were evaluated with the Von Frey Hairs set of 20 nylon monofilaments (Aesthesio^®^, Ugo Basile^®^, Italy) (Deuis et al., 2017; Minett et al., 2011; Chaplan et al., 1994; Bradman et al., 2015; Ferrier et al., 2016; Zumbusch et al., 2024; Cohnstein, 1897). To assess the contribution of collateral sprouting to functional sensory reinnervation, the Von Frey Monofilaments were manually applied to three pre-defined test areas on the reconstructed and non-reconstructed sides considering the sensory innervation of the forepaws (Walcher et al., 2018): The medial (three radial toes - selectively innervated by the median nerve), the lateral (fifth toe – selectively innervated by the ulnar nerve) and the central, overlapping area (fourth toe – non-selectively innervated by afferents of the median and ulnar nerves) (Figure 3). The rats were habituated for over 5 min before the test. The mechanical withdrawal threshold (MWT) in response to a tactile stimulus was determined using a modified up-and down method (Chaplan et al., 1994). The filaments were applied perpendicularly to the glabrous plantar surface of the paw until they bended. Behavior such as flinching, flicking, shaking or licking the paw in response to the application of the filament or immediately after the filament was removed were considered a positive response. This procedure was repeated three times per test area for each filament. If 2 out of 3 applications elicited a positive response, the next lower filament was applied, while lack of response resulted in the application of a filament with a higher bending force. The lowest force required to provoke a positive response was recorded as the MWT (g). The MWT was confirmed by repeating the application of the fiber that elicited the last response.

#### 2.3.2 CatWalk XT

Gait behavior was evaluated by means of the CatWalk XT gait analysis system (Version 10.6, Noldus Information Technology) (Hamers et al., 2001; Heinzel et al., 2020b; Ritter et al., 2023; Timotius et al., 2023; Heinzel et al., 2020a). One week prior to surgeries; animals were familiarized with the test procedure and trained to cross the walkway daily according to an established training protocol (Heinzel et al., 2020b; Ritter et al., 2023). After the end of the training period, all animals were able to perform uninterrupted runs with a speed between 50 and 70 cm/s in accordance with relevant publications (Koopmans et al., 2007). During every session a minimum of three runs per trial were collected (Koopmans et al., 2007; Deumens et al., 2007; Heinzel et al., 2020a). We assessed the following CW parameters according to Deumens classification (Deumens et al., 2007; Table 1): I. General parameters: Print Area (mm^2^), Print Length (mm), Print Width (mm) II. Pain-related parameters: Swing Time (s) and Duty Cycle (%) as well as IV. Other parameters: Swing Speed (cm/s), Stand Index (%) and Single Stance (s). For each parameter we calculated two different ratios: 1. Reconstructed side ratio = Reconstructed front paw/Reconstructed Side hind paw, 2. Non-reconstructed side ratio = Non-reconstructed front paw/Non-Reconstructed Side hind paw. For each postoperative time point these ratios were than compared to the baseline ratios.

**TABLE 1:** Gait parameters assessed with the CatWalk gait analysis system.

Category	Parameter	Explanation
I	Print area (mm^2^)	Total area of a paw print
I	Print length (mm)	Length of a paw print
I	Print width (mm)	Width of a paw print
II	Swing time (s)	Duration of the swing phase of a paw
II	Duty cycle (%)	Percentage of the stand phase in a whole step cycle = (Stand Time/(Stand Time + Swing Time)) x 100
IV	Swing speed (cm/s)	Speed of the paw during the swing phase of the step cycle
IV	Stand index (%)	Speed at which the paw loses contact with the floor
IV	Single stance (s)	Duration of contact with the glass floor with only one paw

I: general, II: pain-related, IV: other parameters of gait.

#### 2.3.3 Reflex based grasping strength

The Grasping Test is a well-established method for assessing the function of the superficial and deep finger flexor muscles, which are predominantly innervated by the median nerve in rats (Stößel et al., 2017; Heinzel et al., 2020c; Bertelli and Mira, 1995; Lauer et al., 2022; Jackson, 1936; Papalia et al., 2003). An apparatus for measuring grasping strength (Grip Strength Meter, Ugo Basile^®^, Italy) was used to conduct the test. For the measurement, the animal was gently held at the base of its tail and approached free-floating to a trapezoidal grasping bar. Once the animal grasped the bar, the experimenter gently lifted the rat by its tail in a smooth and reproducible motion toward the ceiling to elicit the grasping reflex. The grasping bar was fitted with a force transducer connected to a peak amplifier. When the pulling force was applied to the transducer, the display on the electronic unit automatically shows the current grip force. To specifically measure force generated during the grasping motion rather than general forelimb grip strength, we excluded any trail in which the primary force originated from proximal muscles such as the biceps brachii. A trial was therefore considered valid in the absence of flexion of the elbow (biceps muscle) or wrist (flexor carpi ulnaris and flexor carpi radialis muscles). We performed three trials per rat at each evaluation time point. The maximum grasping strength of each trial was recorded. The mean grasping strength was then calculated by averaging the maximum force of all three trials. In addition, we implemented a previously described grip ability rating to assess the return of grip ability (Stößel et al., 2017): 1/3: no observable toe flexion, 2/3: toe flexion with no measurable strength, 3/3: toe with measurable strength.

### 2.4 Wet muscle weight

After the rats were euthanized at WPO12 the FDS muscles of both sides were harvested and weighed with a precision balance (Sartorius A200S, Sartorius GmbH, Göttingen). The muscle weights were normalized to the total body weight of the animal. Muscle harvesting was performed under an operating microscope in a standardized manner. The muscle was exposed along its entire length ensuring that the borders to adjacent muscle as well as the muscle’s origin and insertion were clearly visualized. The FDS muscle was den sharply detached at its origin at the medial epicondyle of the humerus of the rat and distally transected at the level of the carpal tunnel.

### 2.5 Statistical analysis

Statistical analysis was performed with IBM^®^ SPSS^®^ Statistics 29.0.1.1 (IBM). To determine statistically significant differences between the means of all parameters at each postoperative timepoint, the repeated measures analysis of variance (RMANOVA) or the Friedman Test was performed depending on the distribution of data. The level of significance was adjusted using the Bonferroni correction and *p*-values of 0.05 and lower were considered statistically significant. All data were expressed as mean ± standard error of the mean (SEM) and expressed in percent.

To evaluate statistically significant differences of the medians of all parameters between the reconstructed and non-reconstructed sides, the Wilcoxon Signed-Rank Test or the Sign Test was performed for not-normally distributed data. For normally distributed data a paired *t*-test was conducted. All data were calculated and reported as medians with 95% confidence intervals (CI).

For analysis of correlation between CatWalk, Grasping Strength and the Von Frey Test, Spearman’s correlation coefficient was calculated. *p*-values of 0.05 and lower were considered statistically significant. Notably, as Grasping Strength measurements were solely conducted on the reconstructed side, corresponding data for the non-reconstructed side was not available. Consequently, correlation between the Grasping Test and other behavioral data was examined for the reconstructed side ratios, only.

## 3 Results

### 3.1 Animal welfare

All animals recovered from the surgical procedure without any signs of contractures or automutilation on the forepaws. However, at WPO1, one animal demonstrated extensive guarding behavior of the non-reconstructed forepaw during the Von Frey Test. This animal was excluded from further statistical analysis of the Von Frey data (*n* = 9) and correlation analysis (*n* = 9) due to lack of data.

### 3.2 Von Frey test

**FIGURE 4:**
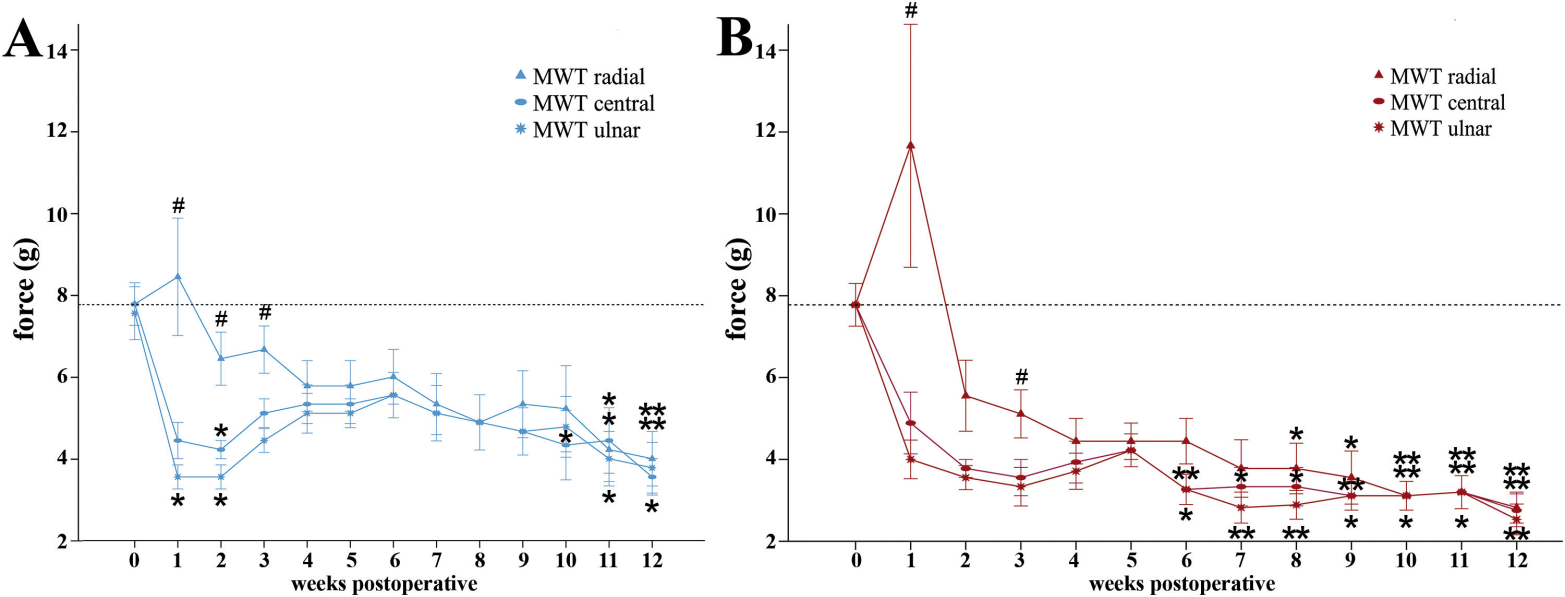
Mechanical withdrawal thresholds as assessed with the Von Frey Test. Time course of the mechanical withdrawal thresholds of the radial, central and ulnar test sites of the reconstructed **(A)** and non-reconstructed **(B)** forepaws from preoperatively (*t* = 0) until 12 weeks after bilateral median nerve injury and unilateral epineurial repair (*n* = 9). *p < 0.05 as compared to Pre-OP; **p < 0.01 as compared to Pre-OP. #*p* < 0.05 radial vs. ulnar; MWT: Mechanical withdrawal threshold. All data were calculated as mean ± standard error of the mean.

On the reconstructed side (Figure 4A) median nerve injury induced a slight decrement of MWT of the radial test site, selectively innervated by the median nerve, to 6.44 ± 0.65 g as early as WPO2. Noteworthy, from WPO6 onward, further gradual decrease of MWT until WPO12 was observable. At WPO11 (4.22 ± 0.78 g, *p* < 0.05) and WPO12 (4.0 ± 0.67 g, *p* < 0.01) rats exhibited significantly different MWTs compared to baseline in this area. In contrast, at the central test site (Figure 4A) of the reconstructed paw, non-selectively innervated by the median and ulnar nerves, rats demonstrated immediate reduction of the MWT at WPO1, reaching a statistically significant minimum (*p* < 0.05) of 4.22 ± 0.22 g at WPO2Pre-OP. Subsequently, from WPO6 onward rats displayed further reduction of MWT until the end of the observation period, consistent with the median nerve innervated area. The MWT was significantly reduced compared to baseline at WPO10 (4.33 ± 0.85 g, *p* < 0.05) and WPO11 (4.44 ± 0.80 g, *p* < 0.05) and reached its lowest value of 3.56 ± 0.44 g at WPO12 (*p* < 0.01). The MWTs of the ulnar test site (Figure 4A) of the reconstructed paw, selectively innervated by the ulnar nerve, revealed a similar course with a statistically significant reduction of MWT from 7.56 ± 0.65 g pre-operatively to 3.56 ± 0.29 g at WPO1 and WPO2 (*p* < 0.05). MWTS further decreased from WPO6 onward revealing statistically significant differences from baseline at WPO11 (4.0 ± 0.67 g, *p* < 0.05) and WPO12 (3.78 ± 0.62 g, *p* < 0.05).

On the radial test site of the non-reconstructed side (Figure 4B), after an initial increase of MWT from 7.78 ± 0.52 g at baseline to 11.67 ± 2.97 g at WPO1, MWT was reduced to 4.44 ± 0.56 g by WPO4. MWTs of the radial test sites were further significantly reduced compared to baseline at later stages between WPO8 and WPO12 with values decreasing from 3.77 ± 0.62 g at WPO8 (*p* < 0.05) until attaining a highly significant minimum of 2.82 ± 0.38 g (*p* < 0.01) at WPO12. Comparable with the reconstructed side, the MWTs the ulnar and central test sites (Figure 4B) were decreased as early as WPO1. Rats exhibited significantly reduced MWTs compared to baseline on both test sites from WPO6 until WPO12 (*p* < 0.01), when maximal change from baseline was observed. Until WPO12, the MWTs had decreased from 4.22 ± 0.40 g at WPO5 to 2.53 ± 0.38 (*p* < 0.01) and 2.76 ± 0.40 g (*p* < 0.01) at the ulnar and central test sites.

Comparison of the MWTs of the different test sides of the reconstructed (Figure 4A) and the non-reconstructed side (Figure 4B) revealed significant (*p* < 0.05) differences between the MWT of the radial and ulnar test site from WPO1 until WPO3 for the reconstructed side and at WPO1 and WPO3 for the non-reconstructed side.

#### 3.2.1 Comparison of the MWTs between the reconstructed and the non-reconstructed sides

**FIGURE 5:**
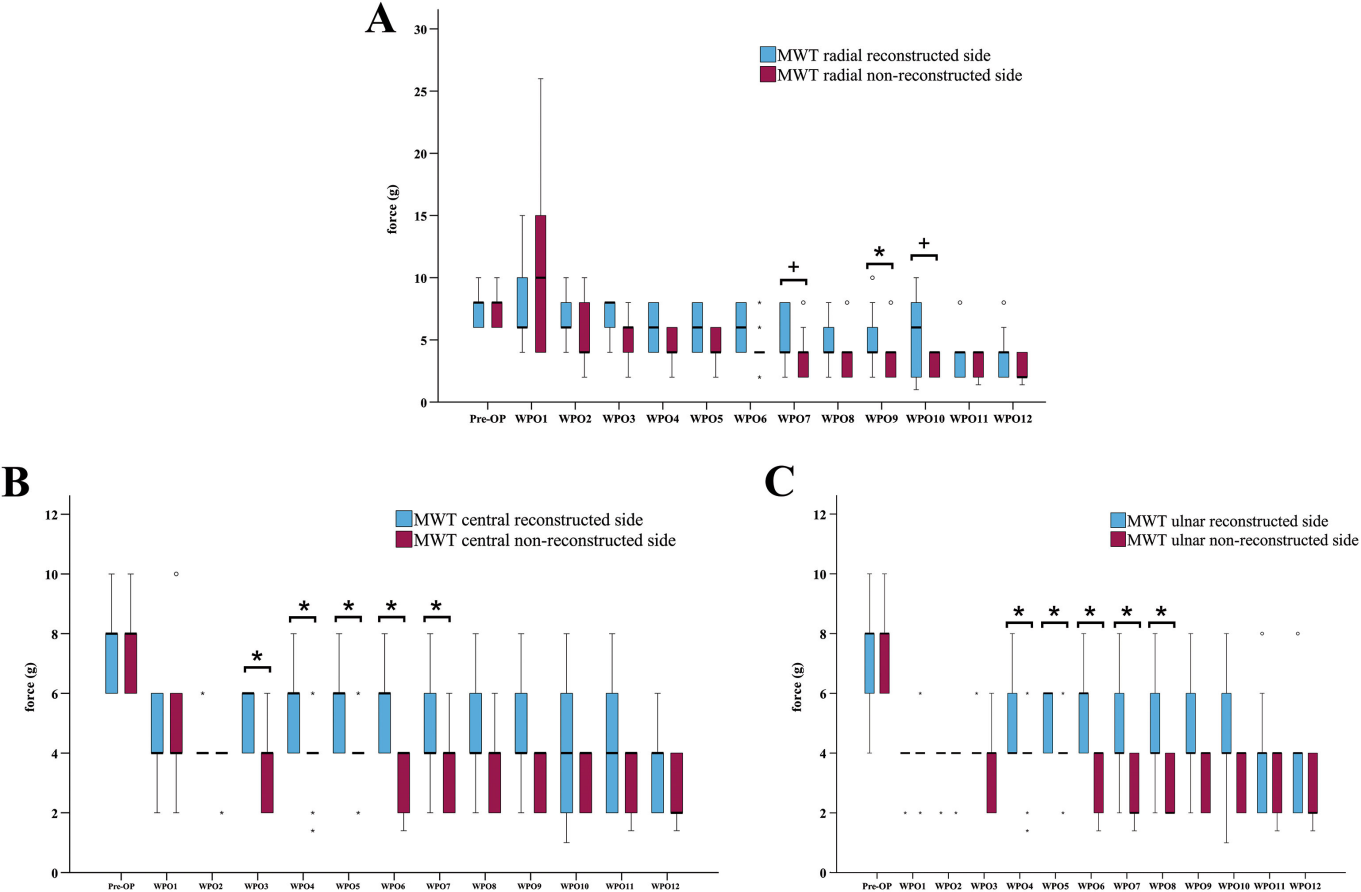
Comparison of the mechanical withdrawal thresholds of the reconstructed and non-reconstructed side. Difference between the mechanical withdrawal thresholds of the radial **(A)**, central **(B)** and ulnar **(C)** test site of the reconstructed and the non-reconstructed sides (*n* = 9). *p < 0.05 reconstructed vs. non-reconstructed side; +*p* < 0.06 indicates almost significant differences; MWT: Mechanical withdrawal threshold, WPO: Week postoperative. Medians are presented with a black line. Stars and dots indicate outliers.

When comparing the MWTs of the radial test site (Figure 5A) between the reconstructed and non-reconstructed side, a statistically significant difference was observable at WPO9 (*z* = 2.271, *p* < 0.05), only. The MWTs of the central test site (Figure 5B) differed significantly between the reconstructed and non-reconstructed side at WPO3 (*z* = 2.333, *p* < 0.05), WPO4 (*z* = 2.333, *p* < 0.05), WPO5 (*z* = 2.236, *p* < 0.05), WPO6 (*z* = 2.414, *p* < 0.05) and WPO7 (*z* = 2.126, *p* < 0.05). Statistically significant difference between the medians of the MWTs of the ulnar test site (Figure 5C) of both sides were observed at WPO4 (*z* = 2.333, *p* < 0.05), WPO5 (*z* = 2.000, *p* < 0.05), WPO6 (*z* = 2.414, *p* < 0.05), WPO7 (*z* = 2.414, *p* < 0.05), WPO8 (*z* = 2.264, *p* < 0.05).

### 3.3 CatWalk gait analysis

#### 3.3.1 Prints of the forepaws

**FIGURE 6:**
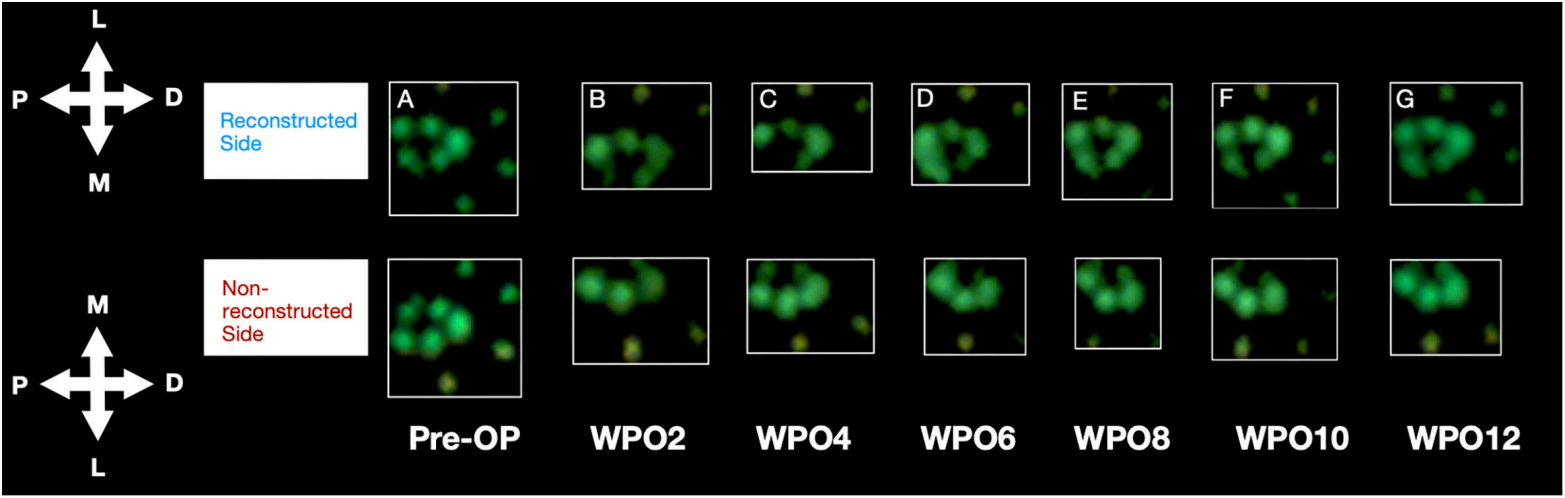
Representative paw prints as assessed with the CatWalk gait analysis system. Paw Prints of the reconstructed and non-reconstructed front paws are shown starting from Pre-OP **(A)** and over the course of the observation period of 12 weeks (biweekly) (**B**–**G**). P: proximal, D: distal, M: medial, L: lateral. WPO = Week postoperative.

Median nerve injury resulted in the rats refraining to bear weight on the medial aspects of the paw and the medial toes, innervated by the median nerve (Figure 6). From WPO6 onward, rats demonstrated increased loading on the medial aspects of the paw on the reconstructed side. At WPO10 and WPO12 increased weight bearing on the medial paw areas was further accompanied by additional loading on the medial toes. In contrast, on the non-reconstructed side weight bearing on the median nerve innervated aspects of the paw remained absent during the entire observation period.

#### 3.3.2 General parameters of gait

##### 3.3.2.1 Print area

**FIGURE 7:**
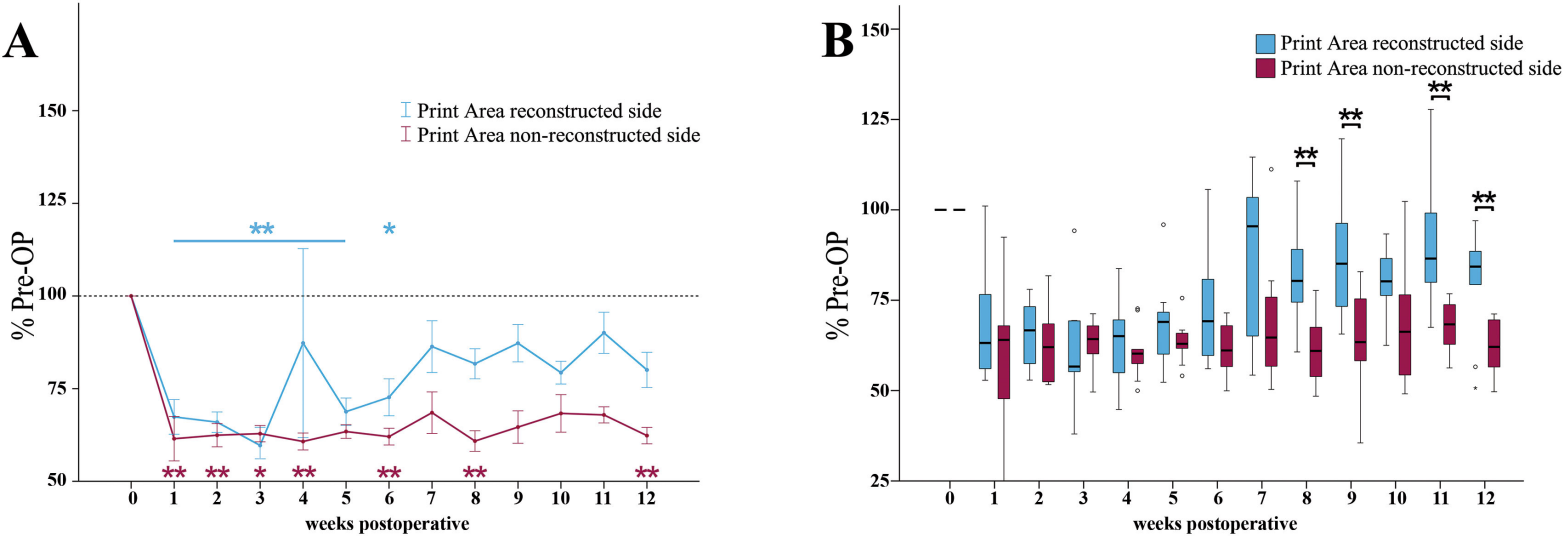
Print area. Course of Print Area of the reconstructed (Reconstructed front paw/Reconstructed side hind paw) and non-reconstructed sides (Non-reconstructed front paw/Non-reconstructed side hind paw) **(A)** from pre-operatively (*t* = 0) until 12 weeks after bilateral median nerve injury and unilateral epineurial repair (*n* = 10). *p < 0.05 as compared to Pre-OP. **p < 0.01 as compared to Pre-OP. All data was calculated as mean ± standard error of the mean and expressed in percent. Comparison of the Print Area medians between the reconstructed and the non-reconstructed sides (*n* = 10) **(B)**. *p < 0.05 as compared to Pre-OP. **p < 0.01 as compared to Pre-OP. Medians are presented with a black line. Stars and dots indicate outliers.

Median nerve injury resulted in a highly significant (*p* < 0.01) reduction of the reconstructed side Print Area ratio (Figure 7A) to 67% of baseline at WPO1, further decreasing to a minimum of 60% by WPO3 (*p* < 0.01). It remained highly significantly (*p* < 0.01) different from baseline until WPO5. From WPO5 onward, it steadily increased to 73% at WPO6, remaining significantly different (*p* < 0.05) from Pre-OP and recovered to 81% of baseline until WPO12.

The non-reconstructed side Print Area ratio (Figure 7A) was highly significantly (*p* < 0.01) reduced to 61% of baseline at WPO1. It reached lowest values at WPO4 (*p* < 0.01) and remained significantly (*p* < 0.01) decreased at WPO6 and WPO8. In contrast to the reconstructed side, it only slightly recovered to 62% of baseline until WPO12, which was highly significantly (*p* < 0.01) different from Pre-OP.

The reconstructed side Print Area Ratio was highly significantly (*p* < 0.01) higher compared to the non-reconstructed side Print Area ratio at WPO8 (*z* = 2.701), WPO9 (*z* = 2.701), WPO11 (*z* = 2.701) as well as WPO12 (*z* = 2.599) (Figure 7B).

##### 3.3.2.2 Print length

The reconstructed side Print Length ratio (Supplementary Figure 1A) was highly significantly (*p* < 0.01) reduced to <80% of baseline at WPO1. The parameter was still significantly reduced from Pre-OP at WPO3 (*p* < 0.05), WPO5 (*p* < 0.05) and WPO6 (*p* < 0.05) and WPO8 (*p* < 0.05). It recovered to values above 82% until WPO12.

On the non-reconstructed side (Supplementary Figure 1A), Print Length ratio was significantly reduced to less than 77% compared to baseline at WPO1 (*p* < 0.05). It reached highly significant minima of 72 and 71% at WPO4 and WPO6 (*p* < 0.01), respectively and remained highly significantly (*p* < 0.01) reduced from Pre-OP until WPO9. In contrast to the reconstructed side, it only slightly recovered and was highly significantly different from baseline until WPO12 (*p* < 0.01)

Significant differences between reconstructed side and non-reconstructed side Print Length ratios were observed at WPO4 (*z* = 2.090, *p* < 0.05), WPO6 (*z* = 2.191, *p* < 0.05) and WPO9 (*z* = 2.701, *p* < 0.01) (Supplementary Figure 1B).

##### 3.3.2.3 Print width

**FIGURE 8:**
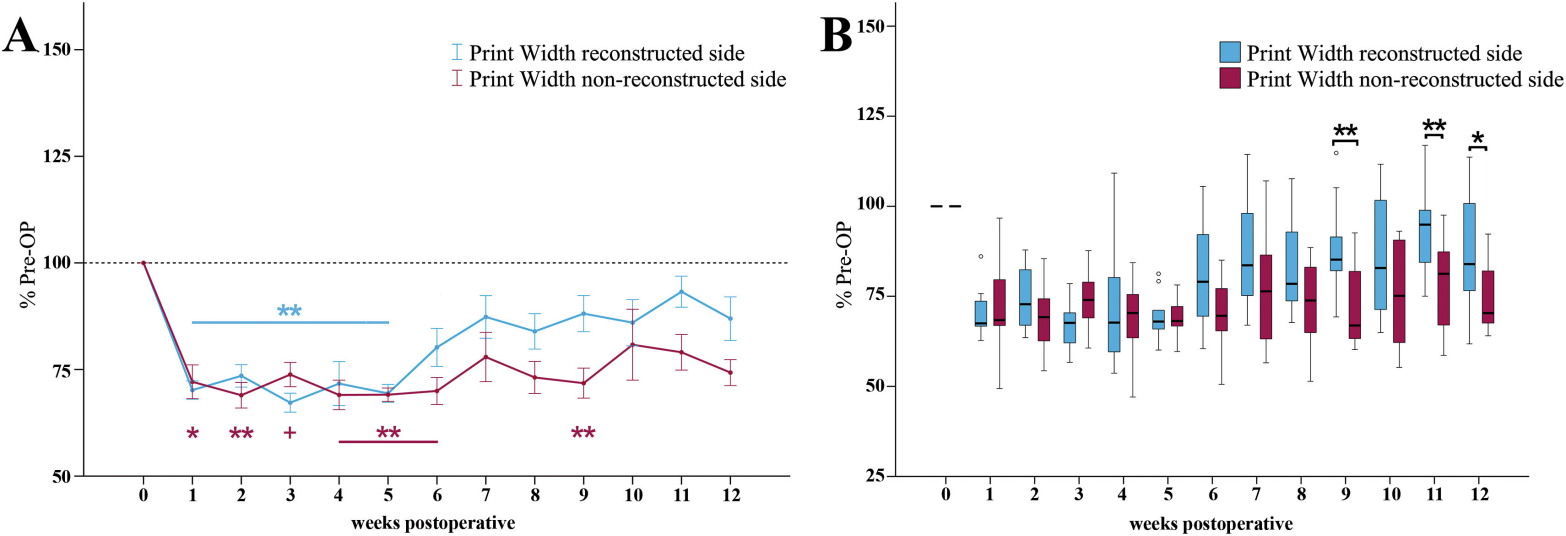
Print width. Course of Print Width of the reconstructed (Reconstructed front paw/Reconstructed side hind paw) and non-reconstructed sides (Non-reconstructed front paw/Non-reconstructed side hind paw) **(A)** from pre-operatively (*t* = 0) until 12 weeks after bilateral median nerve injury and unilateral epineurial repair (*n* = 10). *p < 0.05 as compared to Pre-OP. **p < 0.01 as compared to Pre-OP +*p* < 0.06 indicates almost significant differences compared to Pre-OP. All data was calculated as mean ± standard error of the mean and expressed in percent. Comparison of the Print Width medians between the reconstructed and the non-reconstructed sides (*n* = 10) **(B)**. *p < 0.05 as compared to Pre-OP. **p < 0.01 as compared to Pre-OP. Medians are presented with a black line. Stars and dots indicate outliers.

The reconstructed side Print Width ratio (Figure 8A) was highly significantly (*p* < 0.01) reduced to <74% of baseline in the first five weeks following median nerve injury. It recovered to 86% of baseline until the end of the observation period.

On the non-reconstructed side (Figure 8A), Print Width ratio was significantly (*p* < 0.05) decreased to 72% of baseline at WPO1 and reached a highly significant (*p* < 0.01) minimum of <69% at WPO2. With the exception of WPO3 it was highly significantly (*p* < 0.01) altered and stayed below 70% of baseline until WPO6. It was still highly significantly (*p* < 0.01) different from Pre-OP at WPO9 (*p* < 0.01). In comparison to the reconstructed side, the parameter only slightly recovered to 74% of baseline until WPO12.

Statistically significant differences between the Print Width ratios of the reconstructed and the non-reconstructed side were observed at the later timepoints WPO9 (*z* = 2.803, *p* < 0.01), WPO11 (*z* = 2.599, *p* < 0.01) and WPO12 (*z* = 2.395, *p* < 0.05) (Figure 8B).

#### 3.3.3 Pain-related parameters of gait

##### 3.3.3.1 Swing time

**FIGURE 9:**
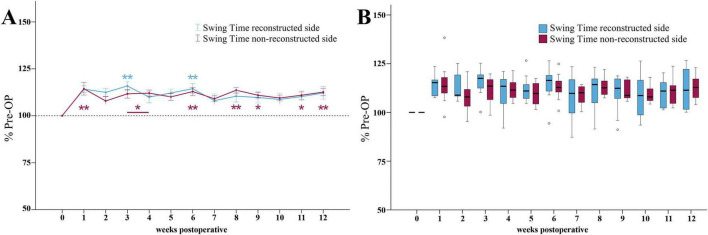
Swing time. Course of Swing Time of the reconstructed (Reconstructed front paw/Reconstructed side hind paw) and non-reconstructed sides (Non-reconstructed front paw/Non-reconstructed side hind paw) **(A)** from pre-operatively (*t* = 0) until 12 weeks after bilateral median nerve injury and unilateral epineurial repair (*n* = 10). *p < 0.05 as compared to Pre-OP. **p < 0.01 as compared to Pre-OP. All data was calculated as mean ± standard error of the mean and expressed in percent. Comparison of the Swing Time medians between the reconstructed and the non-reconstructed sides (*n* = 10) **(B)**. *p < 0.05 as compared to Pre-OP. **p < 0.01 as compared to Pre-OP. Medians are presented with a black line. Stars and dots indicate outliers.

Following median nerve injury, the Swing Time ratio of the reconstructed side (Figure 9A) was increased and reached two maxima of 116 and 114% of baseline at WPO3 and WPO6, which were highly statistically significant (*p* < 0.01) from Pre-OP. From WPO6 onward, the parameter recovered to 112% until the end of the observation period.

The Swing Time ratio of the non-reconstructed side (Figure 9A) was highly significantly (*p* < 0.01) increased to 114% of baseline at WPO1. In contrast to the non-reconstructed side, it remained significantly elevated from baseline at WPO3, WPO4 as well as at later timepoints at WPO9 and WPO11 (*p* < 0.05). It was highly significantly different at WPO6 and WPO8 (*p* < 0.01) as well as at WPO12.

No significant differences in Swing Time ratios between the reconstructed and the non-reconstructed sides were observable (Figure 9B).

##### 3.3.3.2 Duty cycle

**FIGURE 10:**
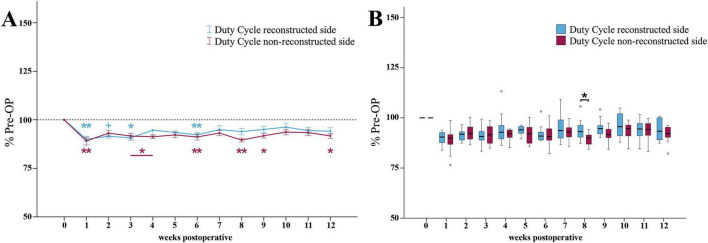
Duty cycle. Course of Duty Cycle of the reconstructed (Reconstructed front paw/Reconstructed side hind paw) and non-reconstructed sides (Non-reconstructed front paw/Non-reconstructed side hind paw) **(A)** from pre-operatively (*t* = 0) until 12 weeks after bilateral median nerve injury and unilateral epineurial repair (*n* = 10). *p < 0.05 as compared to Pre-OP. **p < 0.01 as compared to Pre-OP. +*p* < 0.06 indicates almost significant differences compared to Pre-OP. All data was calculated as mean ± standard error of the mean and expressed in percent. Comparison of the Duty Cycle medians between the reconstructed and the non-reconstructed sides (*n* = 10) **(B)**. *p < 0.05 as compared to Pre-OP. **p < 0.01 as compared to Pre-OP. Medians are presented with a black line. Stars and dots indicate outliers.

At WPO1, the Duty Cycle ratio (Figure 10A) of the reconstructed side was highly significantly (*p* < 0.01) reduced to a minimum of 90% of baseline. It was still highly significantly decreased (*p* < 0.01) compared to Pre -OP at WPO6. However, from WPO6 until WPO12, Duty Cycle recovered to 94%. It was no longer significantly different from Pre-OP.

The Duty Cycle ratio of the non-reconstructed side (Figure 10A) also decreased to a minimum of 89% at WPO1, which was highly significantly (*p* < 0.01) compared to Pre-OP. However, in contrast to the reconstructed side, the Duty Cycle ratio was significantly (*p* < 0.05) altered from baseline not only at the early time points, but also at WPO9 as well as highly significantly (*p* < 0.01) at WPO6 and WPO8 where it reached second and third lowest values. It recovered to 92% by WPO12, which was still significantly different (*p* < 0.05) from Pre-OP.

Between WPO4 and WPO12, the reconstructed side Duty Cycle ratio was slightly increased compared to the non-reconstructed side ratio (Figure 10B). At WPO8, this difference reached statistical significance (*z* = 1.988, *p* < 0.05).

#### 3.3.4 Other parameters of gait

##### 3.3.4.1 Swing speed

The Swing Speed ratio of the reconstructed side (Supplementary Figure 2A) was highly significantly (*p* < 0.01) reduced to around 85% compared to Pre-OP at WPO3 and WPO6. From week 6 onward, the parameter recovered to 89% of baseline until WPO12, which was still significantly different (*p* < 0.05) from baseline.

On the non-reconstructed side, Swing Speed ratio (Supplementary Figure 2A) exhibited a significant reduction (*p* < 0.05) to 87% of baseline at WPO1. The Swing Speed ratio demonstrated highly statistically significant (*p* < 0.01) differences from Pre-OP at WPO4 and from WPO6 until WPO10. It maintained values between 88 and 91% until the end of the observation period and was still highly significantly different from Pe-OP (*p* < 0.01) at WPO12.

There were no statistically significant differences between the Swing Speed ratio of the reconstructed side compared to the non-reconstructed-side until WPO12 (Supplementary Figure 2B).

##### 3.3.4.2 Stand index

**FIGURE 11:**
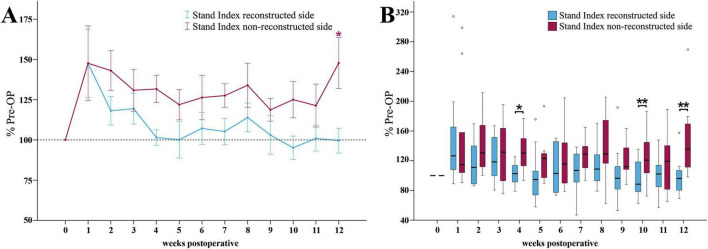
Stand index. Course of Stand Index of the reconstructed (Reconstructed front paw/Reconstructed side hind paw) and non-reconstructed sides (Non-reconstructed front paw/Non-reconstructed side hind paw) **(A)** from pre-operatively (*t* = 0) until 12 weeks after bilateral median nerve injury and unilateral epineurial repair (*n* = 10). *p < 0.05 as compared to Pre-OP. **p < 0.01 as compared to Pre-OP. All data was calculated as mean ± standard error of the mean and expressed in percent. Comparison of the Stand Index medians between the reconstructed and the non-reconstructed sides (*n* = 10) **(B)**. *p < 0.05 as compared to Pre-OP. **p < 0.01 as compared to Pre-OP. Medians are presented with a black line. Stars and dots indicate outliers.

The Stand Index ratio of the reconstructed side (Figure 11A) was increased to 147% of baseline at WPO1. Until the end of the observation period, it recovered to values near 100%. There were no significant differences to baseline at any time point.

The non-reconstructed side Stand Index ratio (Figure 11A) revealed a comparable increase to 148% of baseline at WPO1. In contrast to the reconstructed side, it remained elevated throughout the whole observation period and was significantly different (*p* < 0.05) from Pre-OP at WPO12.

The medians of the reconstructed side Stand Index ratio were significantly lower compared to the non-reconstructed side at WPO4 (*z* = 2.497, *p* < 0.05), WPO10 (*z* = 2.701, *p* < 0.01) and WPO12 (*z* = 2.599, *p* < 0.01) (Figure 11B).

##### 3.3.4.3 Single stance

The reconstructed side Single Stance ratio (Supplementary Figure 3A) revealed an increment following median nerve injury. It was significantly (*p* < 0.05) different from Pre-OP at WPO4 and from WPO8 until WPO10. Single Stance ratio reached a maximum of 112% of baseline at WP12, which was highly significant (*p* < 0.01) compared to Pre-OP.

In accordance with this observation, the non-reconstructed side Single Stance ratio (Supplementary Figure 3A) increased to 110% of baseline at WPO1 and remained increased until WPO12. There were no statistically significant differences compared to Pre-OP throughout the study period.

Single Stance did not exhibit any statistically significant differences between the reconstructed side and the non-reconstructed side ratios (Supplementary Figure 3B).

### 3.4 Reflex based grasping strength

#### 3.4.1 Grasping score

**FIGURE 12:**
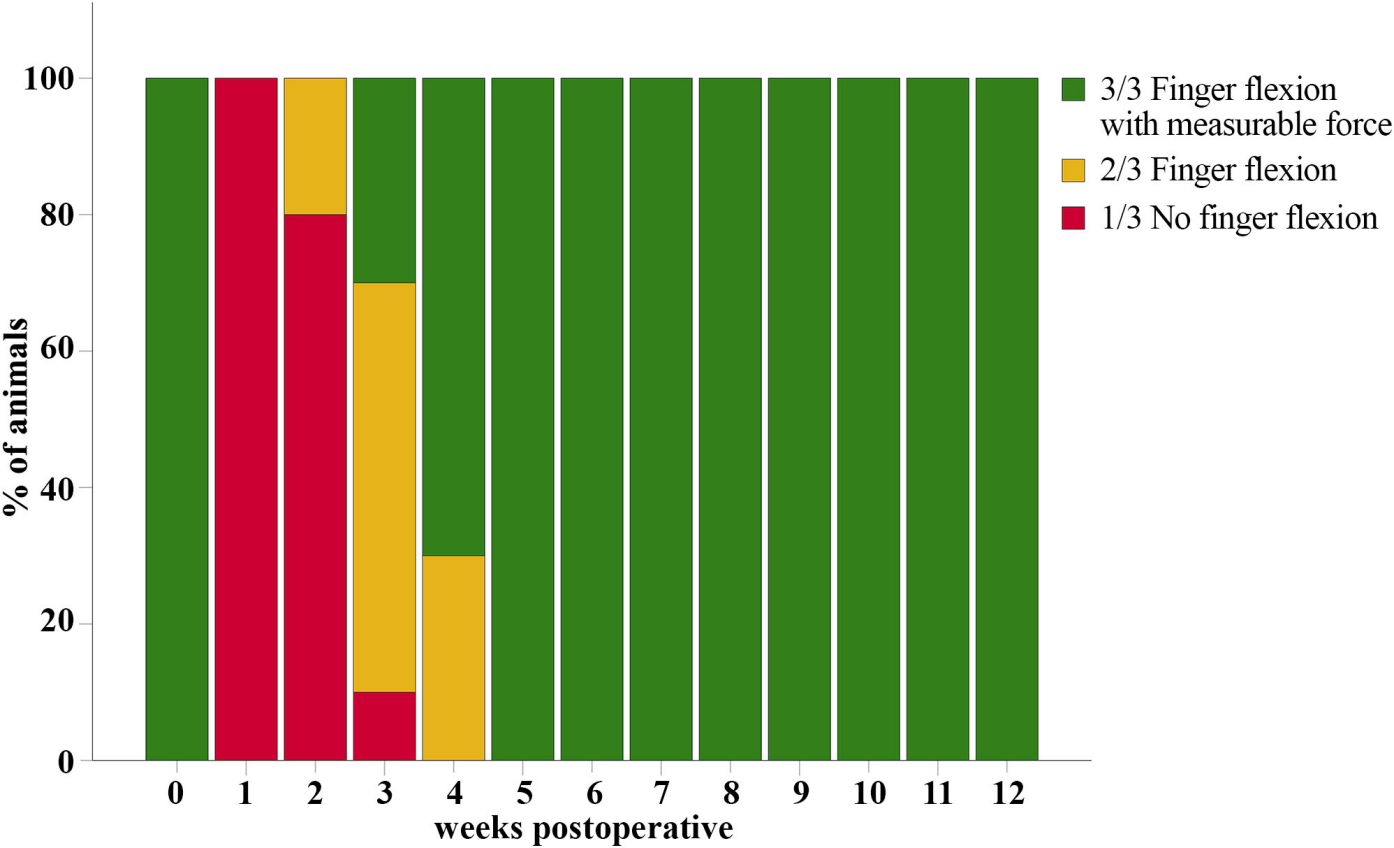
Grasping score. Evaluation of the overall grasping ability prior to (*t* = 0) and after median nerve transection and epineurial repair in rats (*n* = 10). All rats showed reinnervation of the toe flexors and were able to exert measurable grasping strength in the reflex-based Grasping Test at five weeks postoperatively (WPO5).

Median nerve injury resulted in a total loss of overall grasping ability of all rats (*n* = 10) at WPO1 (1/3 on Stossel’s rating scale) (Figure 12). The first signs of motor reinnervation of the finger flexors were evident after 2–3 weeks, with 20% of the rats able to flex the interphalangeal joints in accordance with a Grasping Score of 2/3. Three weeks after injury and repair 30% of the animals had regained the ability to exert a measurable grasping strength. After five weeks, all animals were able to pull the bar with measurable grasping force (3/3).

#### 3.4.2 Mean and max grasping strength

**FIGURE 13:**
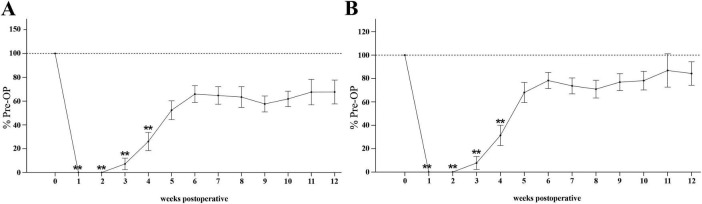
Grasping strength. Time course of mean **(A)** and maximum grasping strength **(B)** of the reconstructed side from pre-operatively (*t* = 0) until 12 weeks postoperatively (*n* = 10). **p < 0.01 as compared to Pre-OP. All data was calculated as mean ± standard error of the mean and expressed in percent.

The Mean Grasping Strength (Figure 13A) was significantly reduced to 0% compared to Pre-OP during the first two weeks after median nerve injury (*p* < 0.01). Subsequently, from WPO2 onward, mean grasping strength progressively increased to 7% at WPO3 and 26% at WPO4, which was still significantly different from Pre-OP (*p* < 0.01). Until WPO12, mean grasping strength recovered to 68% of baseline, no longer displaying statistical significance when compared to Pre-OP data.

The Max Grasping Strength (Figure 13B) followed a similar trend. After the initial significant decrement to 0% of baseline at WPO1 and WPO2 (*p* < 0.01). there was a notable increase to 31% of baseline at WPO4 (*p* < 0.01), still highly significantly different from Pre-OP. Maximum grasping strength recovered to 84% until WPO12, no longer significantly different from baseline.

### 3.5 Wet muscle weight

**FIGURE 14:**
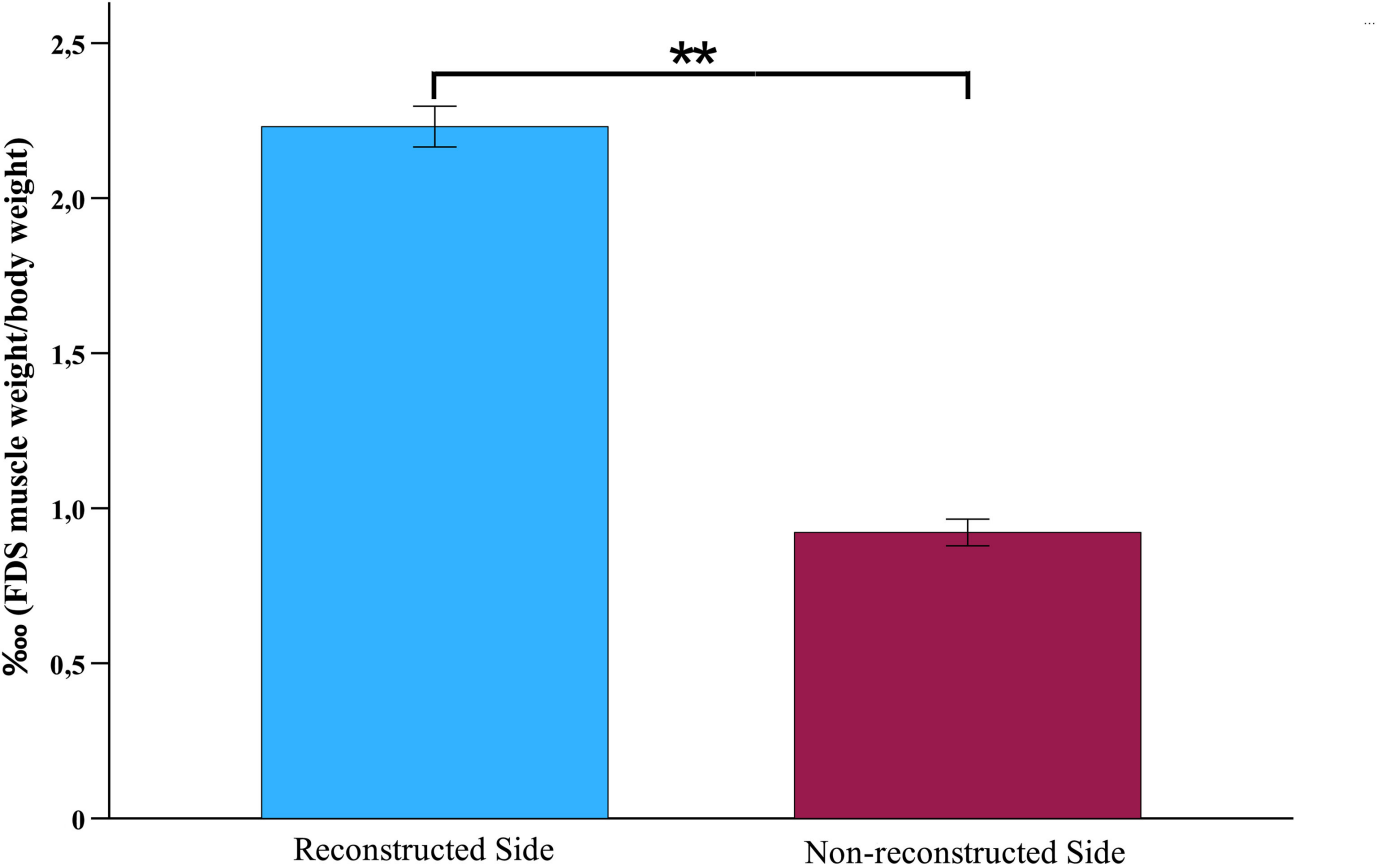
Wet muscle weight. Comparison of the wet muscle weight ratios of the flexor digitorum superficial muscles of the reconstructed and non-reconstructed side at week 12 after median nerve transection and epineurial repair (*n* = 10). Statistical analysis was performed with the parametric paired *t*-test. **p < 0.01 as compared to Pre-OP. FDS: Flexor digitorum superficialis muscle.

**FIGURE 15:**
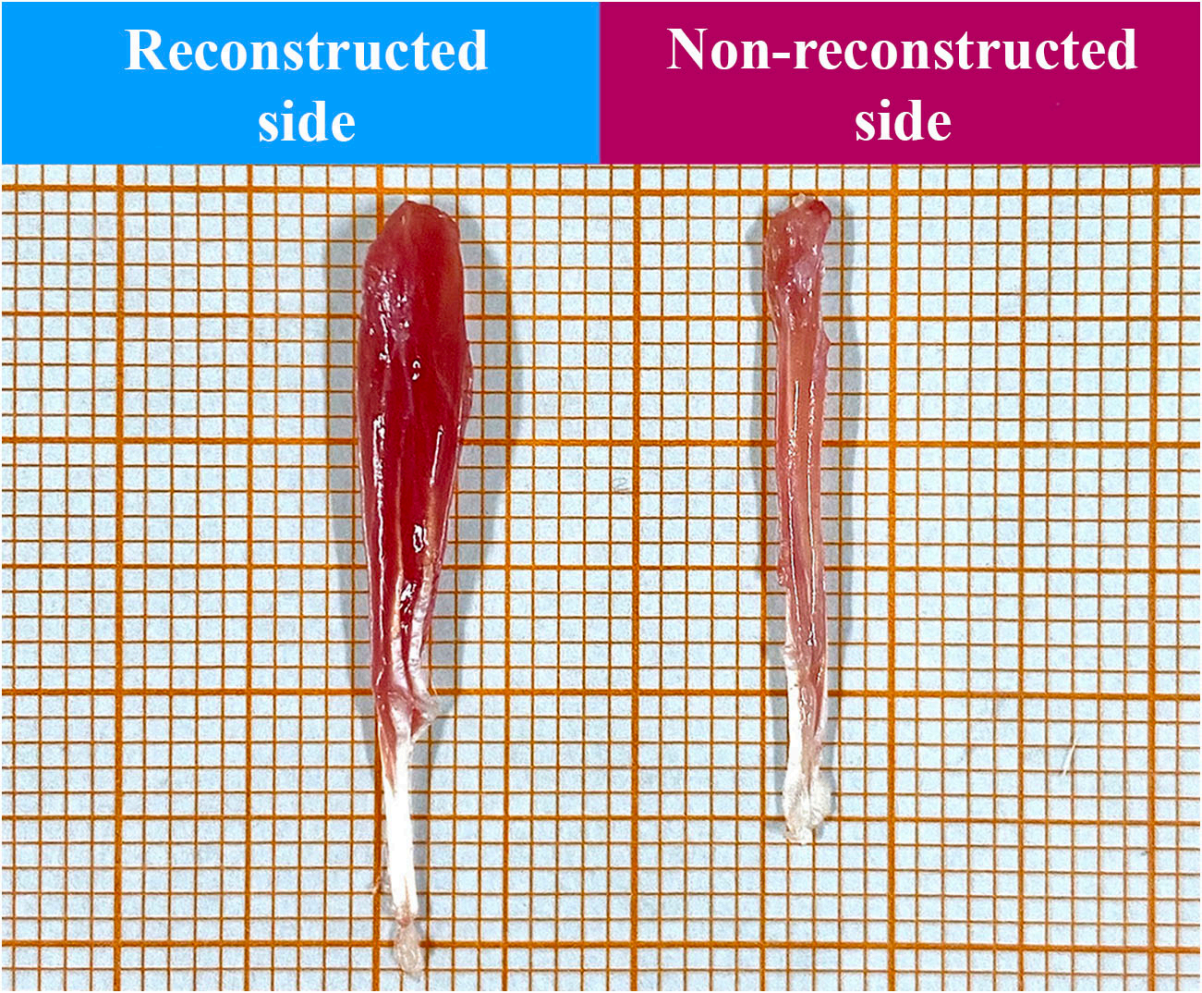
Representative macroscopic appearance of the superficial flexor digitorum muscles of the reconstructed and non-reconstructed side (as indicated) harvested 12 weeks postoperatively.

The ratio of FDS muscle weight to body weight was significantly higher on the reconstructed side (2.23?, SEM = 0.07) than on the non- reconstructed side (0.92?, SEM = 0.04), *t* (9) = 21.906, *p* = < 0.001 (Figures 14, 15).

### 3.6 Correlation analysis

#### 3.6.1 Correlation analysis between CatWalk and Von Frey test data

On the reconstructed side, Print Length showed a significant (*p* < 0.05) correlation with the MWT of the ulnar test site, whereas Print Width (*p* < 0.01) and Print Area (*p* < 0.05) correlated significantly with MWT of the radial test site. The reconstructed side did not reveal any significant correlations between the pain-related parameters of gait and the MWTs as assessed with the Von Frey Test (Supplementary Tables 1 and 2).

On the non-reconstructed side, Print Width correlated significantly (*p* < 0.05) with the MWT of the central test site, while Print Area exhibited a significant (*p* < 0.05) correlation with the MWTs of the central and ulnar test sites (Supplementary Tables 3 and 4).

Additionally, the pain-related parameter Duty Cycle correlated significantly (*p* < 0.05) with the MWT of the ulnar test site. Almost significant correlations were observed between Swing Time and the MWT of the ulnar test sites (*p* = 0.051) as well as between Duty Cycle and the MWT of the central test site (*p* = 0.056). Moreover, there was a significant (*p* < 0.05) correlation between Stand Index and the MWTs of all test sites (Supplementary Tables 3 and 4).

#### 3.6.2 Correlation analysis between CatWalk and grasping test data

Reconstructed side ratios of Print Width, Print Area, Duty Cycle, Swing Speed and Stand Index highly significantly (*p* < 0.01) correlated with Mean and Max Grasping Strength of the reconstructed sides. Swing Time correlated significantly (*p* < 0.05) with Max Grasping Strength and highly significantly (*p* < 0.01) with Mean Grasping Strength. Print Length showed a significant correlation (*p* < 0.05) with Mean Grasping Strength, only (Supplementary Tables 1 and 2).

#### 3.6.3 Correlation analysis between the Von Frey and grasping test data

For the reconstructed side, MWT of the ulnar test site correlated highly significantly (*p* < 0.01) with Mean and Max Grasping Strength (Supplementary Tables 1 and 2).

#### 3.6.4 Correlation analysis within Von Frey data

On the reconstructed and non-reconstructed sides, MWTs of the radial test sites, innervated by the median nerve, correlated highly significantly (*p* < 0.01) with the MWTs of the central, and ulnar test sites, (Supplementary Tables 1 and 2). Additionally, MWT of the central test site revealed a highly significant correlation (*p* < 0.01) with the MWT of the ulnar test site.

#### 3.6.5 Correlations between CW parameters categories

On the reconstructed side, a high degree of correlation (*p* < 0.01) was observable between the general parameters Print Area, Print Width, Print Length and the pain-related parameters Swing Time and Duty Cycle as well as the other parameter of gait Swing Speed (Supplementary Tables 1 and 2). The pain-related parameter Duty Cycle significantly correlated with Stand Index, whereas Swing Time correlated with Single Stance.

Correlation analysis between the distinct CW parameter categories on the non-reconstructed side reveled highly significantly correlations (*p* < 0.01) between Print Area and all pain-related parameters as well as Swing Speed. Print Area correlated significantly (*p* < 0.05) with Stand Index and Single Stance. Print Length correlated highly significantly (*p* < 0.01) with Swing Time and Duty Cycle as well as Swing Speed and significantly (*p* < 0.05) with Stand Index. Print Width correlated significantly (*p* < 0.05) with Duty Cycle and other gait parameters Stand Index and Single Stance. All pain-related parameters highly significantly (*p* < 0.01) correlated with Swing Speed. Moreover, Swing Time correlated significantly with Stand Index (Supplementary Tables 3 and 4).

## 4 Discussion

Due to the limitations of the study regarding the chosen study design and the absence of a separate control group, the present work can only be interpreted as a pilot study and does not allow to draw any conclusions from it. The study represents the first comprehensive evaluation of motor and sensory recovery as well as pain related behavior following median nerve transection and epineurial repair by means of CatWalk gait analysis, the Grasping Test and the Von Frey Test. All three outcome measures are also established in clinical practice, which further highlights their translational value (Moon et al., 2016; Sasaki et al., 2007; Perkins et al., 2010; Bertelli et al., 2018). In addition, our work marks the first investigation of correlations between these three functional tests. Moreover, our pilot study provides novel insights into the sensory reinnervation and the development and maintenance of mechanical allodynia across the distinct innervation areas of the forepaws for the first time in a at upper extremity nerve injury model considering the potential contribution of collateral sprouting.

### 4.1 The Von Frey test

Following median nerve injury, rats demonstrated temporal reduced responsiveness to mechanical stimuli in the areas of the forepaw innervated by the median nerve, a condition that can be attributed to denervation. However, within 2 weeks after injury, the MWTs started to decrease indicating the development of mechanical allodynia. Interestingly, in areas innervated by the uninjured ulnar nerve and the non-selectively innervated areas of the forepaws, signs of early mechanical allodynia were evident as early as one week after injury on both sides. Previous studies reported earliest signs of nerve regeneration in regard to motor function after 2–3 weeks in the rodent median nerve injury model (Lauer et al., 2022; Heinzel et al., 2020c; Daeschler et al., 2018). In contrast, first signs of sensory recovery appeared after more than 4 weeks in a mouse model of median nerve transection and repair (Shaikh et al., 2016). This discrepancy suggests that the early development of mechanical allodynia across all innervation areas in our study, also in areas of the paw, where the nerves are completely intact, may not be solely attributed to axonal regeneration in this early phase after nerve injury, but to other mechanism such as collateral spouting of the adjacent intact ulnar nerve into denervated areas of the forepaw. This hypothesis is further supported by studies demonstrating collateral sprouting of the saphenous and sural nerves as compensatory mechanism in the first weeks after sciatic nerve injury or spared nerve injury. Notably, this early collateral sprouting has been linked to the onset of mechanical allodynia (Cobianchi et al., 2014; Kambiz et al., 2015; Kingery and Vallin, 1989; Devor and Govrin-Lippmann, 1979; Jeon et al., 2024; Kingery et al., 1994; Vallin and Kingery, 1991). A key finding of Cobianchi et al. supporting the contribution of this mechanism, is the surgical transection of the saphenous nerve after 14 days resulting in a complete loss of sensation at the plantar skin of the hindpaw. This observation together with histological data confirmed that the early mechanical allodynia in this model was predominantly mediated by collateral sprouting of the saphenous nerve (Cobianchi et al., 2014). In accordance to these findings, it can be posited that a comparable process might contribute to sensory reinnervation in this model of median nerve injury, especially during the initial phase after nerve injury before axonal regeneration becomes the main driver of median nerve recovery.

In the study at hand, from WPO6 onward, development of late mechanical allodynia was observed in the median nerve innervated areas. This is in a line with findings in the sciatic nerve injury model, where regeneration-induced mechanical allodynia was observed after four weeks. In the same study, transection of the saphenous nerve after 28 days no longer resulted in a loss of sensation at the plantar skin as compared to transection after 14 days, as the sciatic nerve innervated areas and the central areas remained responsive and rats continued to exhibit mechanical allodynia (Cobianchi et al., 2014). This observation strongly supports the conclusion, that mechanical allodynia at this later stage was no longer mediated by collateral sprouting of the saphenous nerve, but rather by axonal regeneration of the sciatic nerve. We therefore hypothesize late mechanical allodynia in our upper extremity model to be indicative of regenerating axons of the median nerve reaching their distal end organs in the skin. Interestingly, several studies reported reduction or even resolution of collateral sprouting induced allodynia once nerve regeneration occurred, supporting the hypothesis that axonal regeneration may counteract maladaptive collateral sprouting (Kingery et al., 1994; Kinnman et al., 1992; Kambiz et al., 2015). However, our results demonstrated long-lasting mechanical allodynia of both forepaws until WPO12 suggesting chronic neuropathic pain. Nevertheless, from WPO6 onward there were significant differences between the reconstructed and non-reconstructed side. Rats experienced reduced mechanical allodynia on the reconstructed side, which we attribute to the reinnervation by regenerating axons of the median nerve. In contrast, on the non-reconstructed side rats exhibited persistent and even aggravated mechanical allodynia, although burying the nerve stumps of the non-reconstructed median nerve in the surrounding muscles, a procedure known to result in smaller, mechanically isolated neuromas (Dellon et al., 1984). We suggest that increased mechanical allodynia on the non-reconstructed side resulted from sustained collateral sprouting of the ulnar nerve, exacerbated by the absence of competing median nerve regeneration on this side. However, persistent mechanical allodynia on the reconstructed side, although alleviated in comparison to the non-reconstructed indicates that both processes, collateral sprouting and nerve regeneration may coexist and interact in a more complex way potentially leading to maladaptive interactions. Further studies are needed to elucidate the underlying mechanism in the development of chronic mechanical allodynia after peripheral nerve injury.

Significant correlations were revealed between the MWTs of the different test sites indicating that reduced MWT of one specific paw area paralleled with decreased MWTs in the other areas. This further supports the hypothesis that collateral sprouting of the intact ulnar nerve paralleled with the development of mechanical allodynia in all test sites of the forepaws. The ulnar nerve may expand its innervation territory through collateral sprouting of axons into adjacent territories in an attempt to reinnervate denervated areas. Although functional recovery after peripheral nerve injury might be achieved by axonal regeneration, by collateral sprouting or to some extent by central neuroplasticity, all of these processes have also been suggested to result in maladaptive alterations and contribute to neuropathic pain (Coderre et al., 1993; Kuner, 2010; Navarro et al., 2007). Several studies have proven that nerve injuries result in unique molecular and neurophysiological alterations in uninjured sensory neurons differing from those in injured sensory neurons. (Hudson et al., 2001; Weissner et al., 2006; Chen et al., 2017; Berta et al., 2017; Djouhri et al., 2012; Djouhri et al., 2006; Yang et al., 2018). Those alterations in uninjured sensory afferents are suggested to play an important role in chronic neuropathic pain (Tran and Crawford, 2020). Collateral sprouting might exacerbate neuropathic pain through hyperinnervation or miswiring of collaterals (Peleshok and Ribeiro-Da-Silva, 2011; Griffin et al., 2010). Nevertheless, the overall mechanisms of collateral sprouting into denervated skin and the contribution to neuropathic pain are still poorly understood. Collateral spouting of uninjured neurons has been suggested to be triggered by exposure to the Wallerian degeneration milieu (Collyer et al., 2014; Bao et al., 2016; Lemaitre and Court, 2021). Wallerian degeneration induces a growth response, which has been stated to also depend on the activation of a unique transcriptional programs resulting in upregulation and overexpression of distinct collateral sprouting associated genes (Lemaitre et al., 2020).

### 4.2 CatWalk gait analysis

Rats exhibited the most prominent gait alterations in the first three weeks after median nerve injury with the rats refraining to bear weight on the median nerve innervated areas comparable with previous studies (Chen et al., 2012a; Chen et al., 2012b; Heinzel et al., 2022; Heinzel et al., 2020c). We suggest, that these early gait alterations are attributed to both sensory and motor dysfunction resulting from median nerve injury. During this time, the MWTs of both sides are significantly reduced, suggesting that this early gait alterations may also be influenced by mechanical allodynia. From WPO4 and 6 onward, gait improvements particularly on the reconstructed side, corresponded with sensory and motor reinnervation by regenerating axons of the median nerve evidenced by recovery of grasping strength and the onset of late mechanical allodynia. Accordingly, significant differences in gait parameters between the reconstructed and non-reconstructed side were evident especially at later time points. Although gait parameters did not fully recover until the end of the observation period, rats exhibited significantly improved gait parameters on the reconstructed side from WPO4 with more robust and evenly paw load on the median nerve innervated areas of the forepaws indicating a significant effect of median nerve repair on functional outcome.

While previous studies have examined correlations between CW parameters and the Von Frey Test after sciatic chronic constriction injury (CCI) or crush injury, our study is the first to demonstrate correlations between sensory and motor function in the median nerve injury model (Vrinten and Hamers, 2003; Hoang et al., 2012; Heinzel et al., 2020c). Significant correlations were identified between general and pain-related gait parameters as well as with the Von Frey data, indicating that static and dynamic gait impairments are influenced not only by motor deficits but also by altered proprioception and pain perception after median nerve injury and repair. A recent meta-analysis demonstrated that standard pharmacological treatments for neuropathic pain, including gabapentinoids, were able to improve gait alterations in preclinical models of traumatic nerve injury (Huerta et al., 2025). These findings support our suggestion that pain contributes to altered gait behavior in addition to motor impairment. Moreover, in a streptozotocin-induced diabetic neuropathy model, CatWalk gait analysis revealed alterations in Print Area, Maximum Contact Area and Stride Length which occurred in parallel with the development of hyperalgesia as assessed with the Von Frey Test (Vieira et al., 2020). These CW parameters correlated strongly with mechanical withdrawal thresholds, further indicating that gait alterations are influenced by neuropathic pain (Vieira et al., 2020). It should be noted, that the CW gait analysis represents a non-evoked behavioral testing method, as the rats traverses the walkway voluntarily. As ambulation is a naturally occurring behavioral pattern, the gait analysis, in contrast to the majority of other methods, is a feasible method to capture non-stimulus evoked pain (Deuis et al., 2017; Ma et al., 2022; Vrinten and Hamers, 2003; González-Cano et al., 2020). A recent systematic review highlighted that gait analysis may be the most effective strategy for measuring non-evoked pain in preclinical models of neuropathic pain and is more likely to reflect clinical presentation of neuropathic pain conditions, which are predominantly characterized by spontaneous, non-evoked rather than stimulus-evoked pain (Huerta et al., 2024).

Bilateral median nerve injury induced an increase of Stand Index and Single Stance (Heinzel et al., 2020c), suggesting the rats attempt to minimize painful contact with the floor and favor less painful limbs, aligning with previous research suggesting Stand Index and Single Stance as potential measures of pain (Kameda et al., 2017; Zahoor et al., 2016; Sakuma et al., 2013; Zhang et al., 2021; Chiang et al., 2014). Chiang et al. identified correlations between Single Stance and expression of Nerve Growth Factor and Tumor Necrosis Factor-alpha in the CCI rat model, supporting its use as a potential pain indicator (Chiang et al., 2014). In our study, the non-reconstructed side consistently exhibited higher Stand Index especially at WPO12, linked to aggravated mechanical allodynia at this timepoint. The correlation between Stand Index and Single Stance with pain-related parameters as well as the inverse correlation of Stand Index and the MWTs of all test sites on the non-reconstructed side as assessed in our study further supported the hypothesis that these gait parameters might be influenced by pain perception (Heinzel et al., 2020c). The lack of correlation between Stand Index and Von Frey data on the reconstructed side might be attributed to reduced mechanical allodynia and the overall increased Print Area on this side due to median nerve regeneration.

### 4.3 Grasping strength

Following median nerve transection and repair rats revealed total loss of grasping ability due to denervation. Earliest signs of motor recovery (flexion of the toes) were observed at WPO2, which was followed by the ability to exert measurable grasping strength starting at WPO3. A systematic review on the grasping test described first signs of motor recovery after a mean of 23.3 days following median nerve transection and epineurial repair while maximal motor recovery was reached after 100 days (Lauer et al., 2022). Daeschler et al did not observed active finger flexion in the first two weeks after median nerve transection and epineurial coaptation in female Sprague Dawley rats, however, grasping strength was measurable at WPO3, which was in a line with our findings (Daeschler et al., 2018). Interestingly, Beck-Broichsitter et al. measured grasping strength as early as 2 weeks after median nerve injury and repair in female rats (Beck-Broichsitter et al., 2014). Several studies have reported more pronounced nerve regeneration in female rats due to neuroprotective effects of the sex hormones (Kovacic et al., 2003; Roglio et al., 2008). In our study, nearly all assessed gait parameters correlated with mean and maximum grasping strength, indicating that gait and grasping strength improvements on the reconstructed side can be attributed to median nerve motor reinnervation. However, although the median nerve predominantly innervates the long toe flexors, contribution of the ulnar nerve to the innervation of this flexor muscles cannot be entirely excluded. Additionally, significant correlations between the MWT of the ulnar test site of the reconstructed side and mean and maximum grasping strength were observable, suggesting that mechanical allodynia might affect grasping strength. In a line, Montilla-García et al. demonstrated in a complete Freund’s adjuvant model of joint inflammation that grip strength can serve as an indirect measure of tactile allodynia, as it correlated with mechanical withdrawal thresholds assessed by the Von Frey Test. Moreover, analgesics such as Oxycodone and Tramadol were able to partially reverse the deficits in grip strength (Montilla-García et al., 2017). To assess grasping strength, it is essential to elicit a grip reflex. Subsequently, the grasping test examines a sensory motor circuit, which might be influenced by altered sensation of the forepaw and neuropathic pain (Lauer et al., 2022; Navarro, 2016; Stößel et al., 2017; Heinzel et al., 2020c).

## 5 Limitations

The present pilot study has several limitations which need to be considered. One important limitation is related to the research design, involving bilateral median nerve injury (reconstructed side vs. non-reconstructed side) within the same animal. On the one hand, this bilateral approach, minimizes the interindividual variability in behavioral outcomes by allowing comparison between the reconstructed and non-reconstructed side within the same animal. Our study design followed previous studies which implemented behavioral analysis in the bilateral median nerve injury models (Dietzmeyer et al., 2019; Sinis et al., 2006; Stößel et al., 2017; Heinzel et al., 2020c). From an ethical perspective, there is evidence that functional deficits after bilateral median nerve injury are mild and not comparable to other nerve injury models such as the sciatic nerve injury model, where even unilateral lesions result in severe motor and sensory impairment (Stößel et al., 2017). Consistently, none of the existing literature on bilateral median nerve injury reported a frequent occurrence of termination criteria due to stress on the animals in the context of bilateral injury (Dietzmeyer et al., 2019; Sinis et al., 2006; Stößel et al., 2017; Heinzel et al., 2020c).

On the other hand, the absence of a separate sham group complicates the distinction between nerve injury-related changes and postoperative pain especially in an early phase after surgery/injury. In our study design, however, functional testing was performed no earlier than one week after surgery, at a time point when no clinical signs of pain were present. Animals received analgesics for the first two postoperative days and thereafter only on demand if pain was observed. We therefore consider it unlikely that postoperative pain had a relevant impact on the observed functional outcomes. Moreover, some might think that the chosen bilateral design might cause confounding effects. In a spinal nerve ligation model it has been demonstrated, that unilateral injury causes changes in the contralateral, uninjured side (Arguis et al., 2008). However specifically for median nerve injury Li et al. reveled that injury to the median nerve on one side only leads to changes in the cortical area of the contralateral median nerve if the ulnar nerve on the same side is also injured, to be the most important and fundamental for our animal experiment design. In this study, an isolated lesion of the median nerve, e.g., on the right side, did not lead to an alteration in the cortex activity of the uninjured median nerve on the uninjured left side as determined by functional magnetic resonance imaging (Li et al., 2013). A bilateral approach, however, might complicate the assignment to condition specific changes (nerve injury with immediate repair/vs. nerve injury without repair). In contrast, unilateral designs with separate sham and control groups might help to distinguish between surgery-related pain, alterations resulting from nerve injury with reconstruction and from nerve injury without reconstruction, but at the cost of substantially increasing the number of animals required. Regarding the implemented functional test there are further specific limitations: The Von Frey Test relies on repeated measurements, which may result in learned behavioral responses, procedural sensitization and increased stress. To minimize these effects, the number of applications of the Von Frey filaments should be reduced, if possible, and sufficient rest between trials should be provided. In addition to methodological limitations, future studies should consider sex differences in the evaluation of nerve regeneration and neuropathic pain following peripheral nerve injury. Recent studies suggest, that female rats are more prone to developing mechanical allodynia after peripheral nerve injury than male rats, likely due to ovarian hormones and genetic factors (Coyle et al., 1996; Dominguez et al., 2008). Moreover, female rats appear to exhibit more extensive collateral sprouting potentially attributed to a higher sprouting capacity of thin myelinated sensory axons (Kovacic et al., 2003). A further limitation of the present pilot study is the lack of histological analyzes of the median nerves and the FDS muscles, that would support the observed behavioral phenomena und would allow for comprehensive assessment of muscle atrophy and reinnervation. Advanced techniques, such as retrograde labeling and immunohistochemistry, should be incorporated and correlated with functional outcomes measures in future studies to distinguish between axonal regeneration and collateral sprouting and to characterize distinct patterns of cutaneous reinnervation associated with each mechanism. Based on our pilot study findings, future studies should also consider transecting the ulnar nerve at various timepoints after median nerve injury to confirm and elucidate the contribution of collateral sprouting to mechanical allodynia, in a line with previous work in the sciatic nerve model (Cobianchi et al., 2014).

## 6 Conclusion

Median nerve injury resulted in persistent functional deficits and mechanical allodynia across all innervation territories of the forepaws. Early mechanical allodynia in median nerve innervated areas may indicate a contribution of compensatory collateral sprouting from the adjacent intact ulnar nerve, whereas late allodynia is more likely attributed to median nerve regeneration. Both processes seem to contribute to sensory reinnervation and the persistence of mechanical allodynia. These results highlight the importance of considering the specific innervation territories when assessing sensory function in peripheral nerve injury models. The CatWalk gait analysis, the Grasping Test and the Von Frey Test proved to be feasible methods not only for evaluating motor recovery, but also for identifying different sensory reinnervation patterns in the rat median nerve injury model. Understanding the distinct contribution and interaction of collateral nerve sprouting and axonal regeneration after nerve injury to the development of functional deficits and chronic neuropathic pain will be crucial in the search of future therapeutic strategies.

## Data Availability

The original contributions presented in this study are included in this article/Supplementary material, further inquiries can be directed to the corresponding author.
