# Supplementary material for: The course of mechanical allodynia differs between forelimb innervation territories following median nerve injury in the rat
Source: Front Neurosci. 2025 Sep 19;19:1602524. doi: 10.3389/fnins.2025.1602524 (PMC12491299; doi:10.3389/fnins.2025.1602524)
Supplement: Supplementary file 1 [file Data_Sheet_1.pdf]

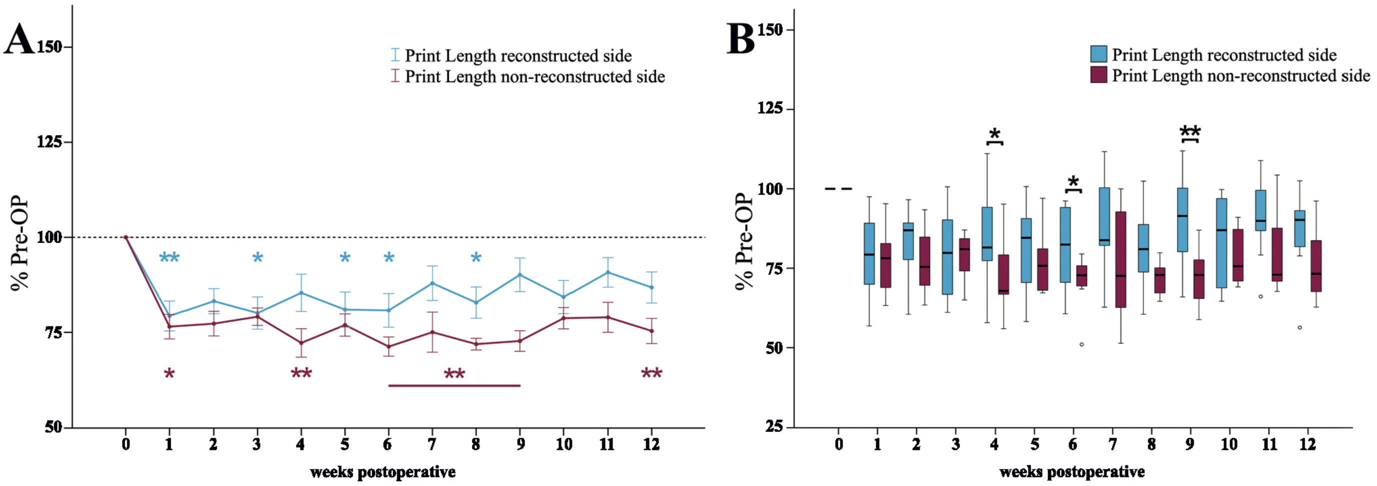

Supplementary Fig. 1. Print Length. Course of Print Length of the reconstructed (Reconstructed side front paw / Reconstructed side hind paw) and non-reconstructed sides (Non-reconstructed side front paw/Non-reconstructed side hind paw) (A) from preoperatively (t=0) until 12 weeks after bilateral median nerve injury and unilateral epineurial repair (n=10). *p < 0.05 as compared to Pre-OP. **p < 0.01 as compared to Pre-OP. All data was calculated as mean ± standard error of the mean and expressed in percent. Comparison of the Print Length medians between the reconstructed and the non-reconstructed sides (n=10) (B). *p < 0.05 as compared to Pre-OP. **p < 0.01 as compared to Pre-OP. Medians are presented with a black line. Stars and dots indicate outliers.

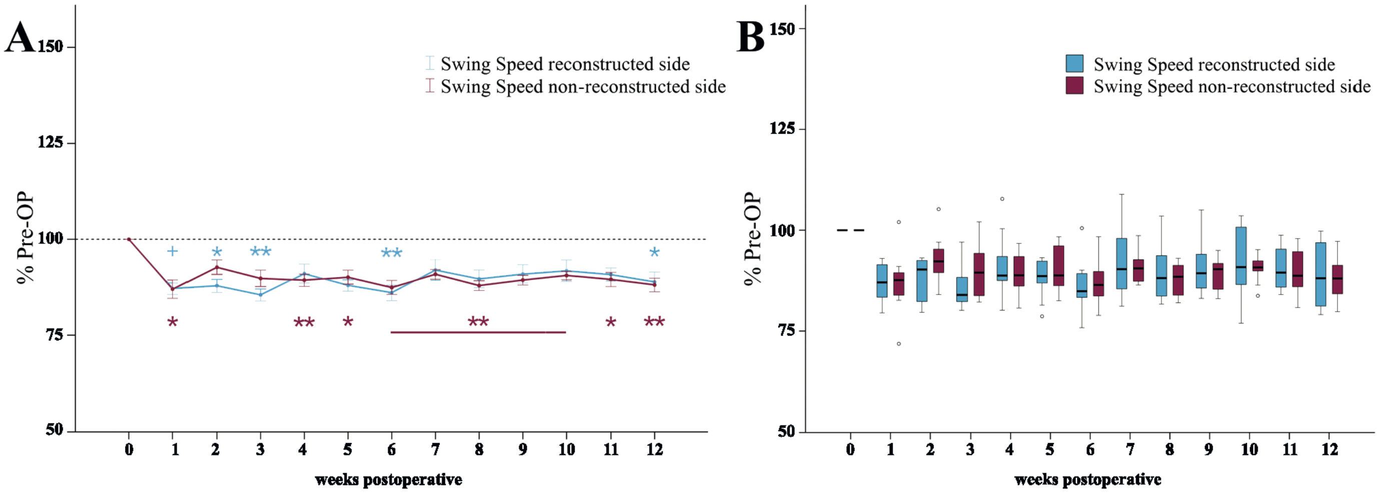

Supplementary Fig. 2. Swing Speed. Course of Swing Speed of the reconstructed (Reconstructed side front paw/Reconstructed side hind paw) and non-reconstructed sides (Non-reconstructed side front paw/Non-reconstructed side hind paw) (A) from preoperatively (t=0) until 12 weeks after bilateral median nerve injury and unilateral epineurial repair (n=10). *p < 0.05 as compared to Pre-OP. **p < 0.01 as compared to Pre-OP. +p<0.06 indicates almost significant differences compared to Pre-OP. All data was calculated as mean ± standard error of the mean and expressed in percent. Comparison of the Swing Speed medians between the reconstructed and the non-reconstructed sides (n=10) (B). *p < 0.05 as compared to Pre-OP. **p < 0.01 as compared to Pre-OP. Medians are presented with a black line. Stars and dots indicate outliers.

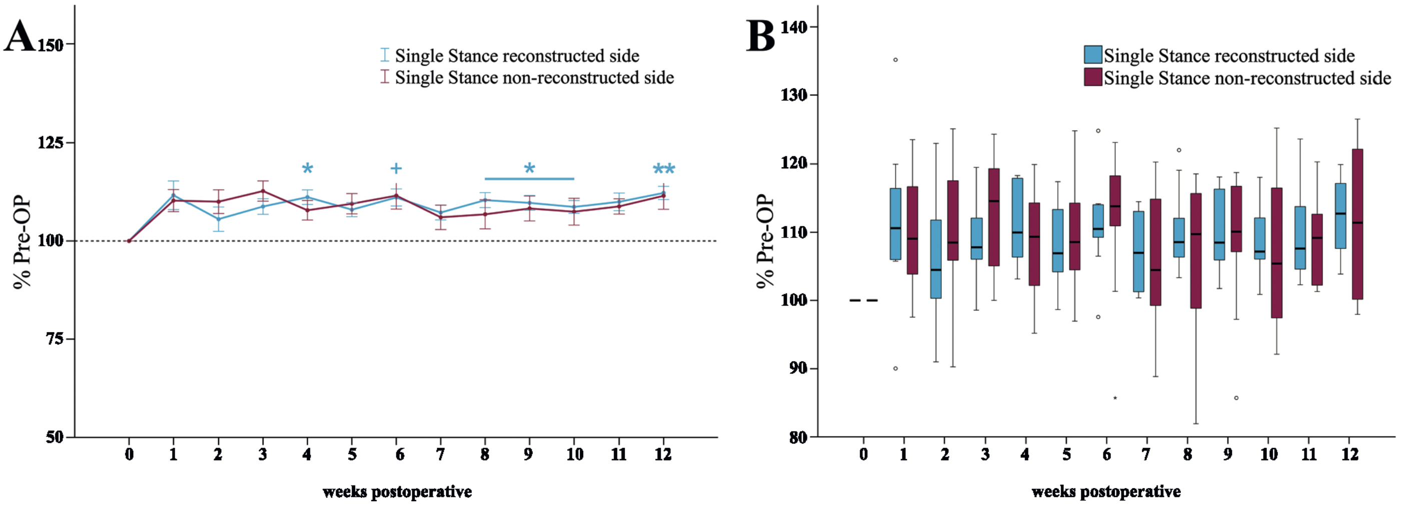

Supplementary Fig. 3. Single Stance. Course of Single Stance of the reconstructed (Reconstructed side front paw/Reconstructed side hind paw) and non-reconstructed sides (Non-reconstructed side front paw/Non-reconstructed side hind paw) (A) from preoperatively (t=0) until 12 weeks after bilateral median nerve injury and unilateral epineurial repair (n=10). *p < 0.05 as compared to Pre-OP. **p < 0.01 as compared to Pre-OP. +p<0.06 indicates almost significant differences compared to Pre-OP. All data was calculated as mean ± standard error of the mean and expressed in percent. Comparison of the Single Stance medians between the reconstructed and the non-reconstructed sides (n=10) (B). *p < 0.05 as compared to Pre-OP. **p < 0.01 as compared to Pre-OP. Medians are presented with a black line. Stars and dots indicate outliers.
